# Genome-wide analysis and growth-promoting potential of *Burkholderia gladioli* YNK-FB0053

**DOI:** 10.3389/fmicb.2026.1858481

**Published:** 2026-07-09

**Authors:** Dexian Wu, Yan Chen, Te Pu, Zhufeng Shi, Weihua Pei, Jiacai Tang, Jinbi Hu, Qibin Chen, Peiwen Yang

**Affiliations:** 1 College of Plant Protection, Yunnan Agricultural University, Kunming, China; 2 Institute of Agricultural Environment and Resources, Yunnan Academy of Agricultural Sciences, Kunming, China; 3 Agricultural and Rural Development Service Center, Yuxi, Yunnan, China

**Keywords:** *Burkholderia gladioli*, plant growth promotion, rhizosphere colonization, soil nutrient mobilization, sulfur-oxidizing bacteria, whole-genome analysis

## Abstract

**Introduction:**

Sulfur-oxidizing bacteria can contribute to the conversion of reduced or organic sulfur pools into plant-available sulfate, thereby improving soil sulfur availability and supporting sustainable agricultural production. This study aimed to characterize the whole-genome features of the rapeseed rhizosphere strain *Burkholderia gladioli* YNK-FB0053, clarify its predicted genetic potential related to sulfur metabolism, nutrient mobilization, rhizosphere adaptation, and plant growth promotion, and validate selected functional traits through phenotypic and pot experiments.

**Methods:**

Whole-genome sequencing of strain YNK-FB0053 was performed using combined Illumina NovaSeq short-read sequencing and PacBio Sequel third-generation single-molecule long-read sequencing, followed by genome assembly, quality assessment, and multi-database functional annotation. The complete genome sequence has been deposited in the GenBank database under accession number JBQPHS000000000. Assembly quality was evaluated using sequencing depth, read-mapping coverage, CheckM completeness/contamination, and BUSCO completeness. Genes predicted to be involved in sulfur metabolism, secondary metabolite biosynthesis, nutrient transformation, phytohormone production, and rhizosphere colonization were systematically identified. Biofilm formation assays, root colonization tests, and Chinese cabbage pot experiments were conducted to evaluate rhizosphere adaptability and plant growth-promoting effects.

**Results:**

The final assembly consisted of two circular chromosomes totaling 8,148,635 bp, with a GC content of 68.07%. Illumina sequencing generated 996,434,009 bp of clean data with Q20 and Q30 values of 97.82 and 90.65%, respectively, while PacBio sequencing generated 548,213,285 bp of reads with an average read length of 10,518.29 bp and a reads N50 of 10,547 bp. The PacBio sequencing depth was 67.28 × , and both Illumina and PacBio reads showed 100% read-mapping coverage of the assembly. CheckM estimated genome completeness as 100%, with 1.68% contamination and 0% strain heterogeneity, while BUSCO analysis showed 100% complete single-copy BUSCOs. A total of 6,898 protein-coding genes were predicted, with an average coding gene length of 1,022.80 bp. Sulfur metabolism annotation identified sqr, Sox-related genes (*soxA, soxB, soxC, soxD*, and *soxG*), the *cysPUWA* sulfate/thiosulfate transport system, assimilatory sulfate reduction genes, and organic sulfur utilization genes, indicating a genetic basis for reduced sulfur oxidation, thiosulfate-related transformation, sulfate assimilation, and diverse sulfur-source utilization. In addition, genes associated with inorganic nitrogen transformation and assimilation, phosphate mobilization, zinc homeostasis, iron acquisition and siderophore-related processes, indole-3-acetic acid biosynthesis, biofilm formation, rhizosphere colonization, phenolic compound degradation, heavy metal resistance, and antibiotic resistance-related traits were also identified. Phenotypic assays showed that the strain formed wrinkled biofilms on MSgg medium within 24 h, with a biomass of 131.4 mg. After 3 days of inoculation, root colonization reached 3.8 × 10^9^ CFU/g. Pot experiments showed that inoculation with YNK-FB0053 significantly increased SPAD value, plant height, and biomass of Chinese cabbage by 15–31%, and also improved plant and rhizosphere nutrient status.

**Conclusion:**

*Burkholderia gladioli* YNK-FB0053 carries multiple genes predicted to participate in sulfur metabolism, nutrient mobilization, iron acquisition and siderophore-related processes, phytohormone biosynthesis, biofilm formation, and rhizosphere adaptation. Among these, *sqr*, Sox-related genes, sulfate/thiosulfate transport genes, assimilatory sulfate reduction genes, and organic sulfur utilization genes provide a focused genomic basis for its sulfur-related functional potential. Phenotypic assays confirmed its biofilm formation and root colonization ability, and pot experiments demonstrated that inoculation with YNK-FB0053 promoted the growth and nutrient status of Chinese cabbage under controlled greenhouse conditions. Together, these genome-based predictions and phenotypic results support YNK-FB0053 as a multifunctional plant growth-promoting bacterial resource with sulfur-related biofertilizer potential. However, genome-based functional predictions, particularly those related to nitrogen metabolism, iron acquisition and siderophore-related traits, phytohormone biosynthesis, antibiotic resistance, and biosafety, require additional targeted biochemical validation and risk assessment before field-scale agricultural application.

## 1 Introduction

Sulfur (S) is an indispensable fourth major macronutrient for plant growth and development. It participates in the biosynthesis of proteins, vitamins, and key enzymes, which is critical for regulating the yield and quality of economic crops such as oil crops. Meanwhile, sulfur promotes the uptake and utilization of nitrogen, phosphorus, iron, and other nutrients, maintains normal plant physiological metabolism, and enhances stress resistance (Li et al., 2020; Narayan et al., 2023; Zenda et al., 2021). Currently, factors including intensive cropping systems, reduced atmospheric sulfur deposition, and insufficient application of sulfur-containing fertilizers have led to a continuous decline in soil sulfur availability worldwide. Sulfur deficiency in crops has become increasingly common, especially in tropical and subtropical regions, posing a major bottleneck restricting sustainable agricultural development (Sharma et al., 2024). Most sulfur in soil exists in organic forms that are barely available to plants; it must be converted to plant-available sulfate through microbial mineralization/desulfonation and oxidation of reduced or elemental sulfur (Gahan and Schmalenberger, 2014; Kertesz and Mirleau, 2004). Therefore, screening high-efficiency and multifunctional sulfur-oxidizing bacteria (SOB) and clarifying their growth-promoting molecular mechanisms are key strategies to alleviate sulfur deficiency stress and reduce reliance on chemical fertilizers (Chaudhary et al., 2023; Ranadev et al., 2023).

*Burkholderia* is a Gram-negative genus widely distributed in soil and plant rhizosphere, belonging to beta-Proteobacteria. It possesses a relatively large genome and strong environmental adaptability. Some plant-associated strains exhibit multiple plant growth-promoting traits, including sulfur oxidation, phosphorus solubilization, siderophore secretion, and antimicrobial activity, making them potential candidates for microbial inoculant development (Mannaa et al., 2019; Yin et al., 2025). However, members of this genus include both beneficial environmental strains and opportunistic pathogens, so biosafety assessment is necessary before agricultural application (Depoorter et al., 2016; Eberl and Vandamme, 2016). In our previous study, a highly active sulfur-oxidizing bacterial strain, YNK-FB0053, was isolated from the rhizosphere soil of oilseed rape in Yunnan, China, and identified as *Burkholderia gladioli*. The strain produced 38.53–66.54 mg/L sulfate and showed zinc solubilization, phosphate solubilization, and siderophore-producing activities. It also grew on nitrogen-free medium, although this phenotype alone does not demonstrate biological nitrogen fixation. The strain improved seed germination and seedling growth of oilseed rape, tomato, and rye and promoted plant growth and nutrient accumulation in tomato pot experiments, indicating agricultural application potential (Chen et al., 2026).

Although partial growth-promoting activities of YNK-FB0053 have been verified, its genome-wide functional characteristics based on COG, GO, KEGG, CAZy, CARD, and antiSMASH annotations remain insufficiently clarified. In particular, the genetic basis associated with sulfur metabolism, nitrogen assimilation and transformation, phosphorus mobilization, rhizosphere colonization, and stress adaptation requires systematic analysis. Because the presence of a gene does not necessarily demonstrate expression or phenotypic activity, genome annotation should be interpreted as predicted functional potential unless supported by independent biochemical, physiological, or plant-assay evidence. Biofilm formation and rhizosphere colonization are essential prerequisites for sustained plant growth promotion by microorganisms (Santoyo et al., 2021; Ajijah et al., 2023; Rafique et al., 2024). Thus, characterizing these traits is vital for inoculant development and field application. Furthermore, the growth-promoting suitability of this strain on other crops such as Chinese cabbage has not been validated, and the uniqueness of its growth-promoting pathways remains to be confirmed. In addition, previous tomato pot experiments did not analyze changes in soil physicochemical properties or consider the effects of sulfur fertilization on the functional performance of this strain.

Accordingly, this study performed whole-genome sequencing and multi-database functional annotation of YNK-FB0053 to clarify its genome features and to identify genes and pathways predicted to participate in sulfur oxidation, sulfur transport, sulfate assimilation, nutrient transformation, and rhizosphere adaptation. Particular attention was paid to the sulfur-related genetic architecture, including *sqr*, Sox-related genes, sulfate/thiosulfate transport systems, assimilatory sulfate reduction genes, and organic sulfur utilization genes. Biofilm formation and rhizosphere colonization ability were determined to validate key traits related to rhizosphere adaptation. Using Chinese cabbage (a Brassicaceae crop) as the model plant, we designed treatments with and without sulfur fertilizer and with different bacterial concentrations to systematically evaluate the effects of this strain on agronomic traits, nutrient contents, and soil physicochemical properties. Correlation and principal component analyses were applied to explore possible growth-promoting mechanisms. This study aims to enrich the sulfur-oxidizing bacterial strain resource library, provide a genome-based interpretation of the sulfur-related multifunctional potential of YNK-FB0053, and offer a reference for the functional analysis and safety-oriented application of sulfur-oxidizing *Burkholderia* strains.

## 2 Materials and methods

### 2.1 Experimental materials

#### 2.1.1 Strains and plant materials

Test strain: *Burkholderia gladioli* YNK-FB0053 was isolated from the rhizosphere soil of rapeseed in Yunnan Province, China, deposited in the China Center for Type Culture Collection (CCTCC NO: M20242288), and associated with the patent number ZL 2024 1 1827636.3. The strain was revived on NA plates, and a single colony was inoculated into nutrient broth (NB) medium and cultured at 28°C and 180 r/min for 24 h as the seed culture for subsequent sequencing, colonization, and pot experiments. The isolation, purification, and preliminary plant growth-promoting phenotypic characterization of this strain were described in our previous study (Chen et al., 2026).

Test plant: Cruciferous Chinese cabbage (*Brassica rapa* subsp. *pekinensis cv. Xiayang 303*).

Test soil: Soil was collected from an oilseed rape field in Yangqiao Township, Songming County, Kunming City, Yunnan Province, China (103.03°E, 25.30°N). After sampling, stones, plant residues, and other impurities were removed, and the soil was thoroughly homogenized, air-dried, and passed through a 2 mm sieve before use in the pot experiment. All treatments used the same homogenized batch of soil, and the experiment was designed to compare treatment-induced differences in plant growth and rhizosphere soil physicochemical properties relative to the sterile-water and NB medium controls under a uniform soil background.

Test fertilizer: Elemental sulfur fertilizer with a purity of 84%.

#### 2.1.2 Culture media

All media used in this study were sterilized by autoclaving at 121°C for 30 min. The medium formulations were as follows:

Nutrient Broth (NB) medium (g/L): Beef extract 3.0 g, peptone 10.0 g, NaCl 5.0 g, pH 7.4.

NA medium (g/L): Peptone 10.0, beef powder 3.0, sodium chloride 5.0, agar 15.0, pH 7.30 ± 0.12.

MSgg medium: 100 mmoL/L MOPS, 5 mmoL/L potassium phosphate (K_2_HPO_4_:KH_2_PO_4_ = 1:1), 2 mmoL/L MgCl_2_, 700 μmoL/L CaCl_2_, 50 μmoL/L MnCl_2_, 50 μmoL/L FeCl_3_, 1 μmoL/L ZnCl_2_, 2 μmoL/L thiamine, 0.5% (m/v) glycerol, 0.5% (m/v) glutamic acid, 50 μg⋅mL^–1^ tryptophan, 50 μg⋅mL^–1^ phenylalanine, 50 μg⋅mL^–1^ threonine, pH 7.0 (Beauregard et al., 2013; Shen, 2024).

### 2.2 Experimental methods

#### 2.2.1 Whole-genome sequencing and assembly

*Burkholderia gladioli* YNK-FB0053 was inoculated into NB medium and cultured at 28°C and 180 r/min for 24 h. Bacterial cells were collected by centrifugation at 10,000 rpm for 15 min, and high-quality genomic DNA was extracted for whole-genome shotgun sequencing. Raw Illumina reads were quality-filtered using fastp v0.20.0. Illumina paired-end libraries and PacBio SMRT long-read libraries were constructed. Short-read sequencing was performed on the Illumina NovaSeq platform using a 2 × 150 bp strategy, whereas long-read sequencing was performed on the PacBio Sequel third-generation single-molecule sequencing platform. After quality control, sequencing reads were used for genome assembly. Genome assembly was performed using hifiasm v0.16.1, Flye v2.9.2, and Unicycler v0.4.8, and the assembled genome was corrected with high-quality Illumina reads using Pilon. Assembly quality was evaluated using clean data statistics, Q20/Q30 values, sequencing depth, read-mapping coverage, k-mer-based genome coverage, CheckM completeness and contamination, and BUSCO completeness (BUSCO v5.4.5).

#### 2.2.2 Genome annotation and functional gene analysis

Genome annotation was performed using a reproducible multi-step pipeline. Tandem repeats were predicted using Tandem Repeats Finder v4.09.1, and interspersed repeats were identified using RepeatMasker v4.1.5. tRNA genes were predicted with tRNAscan-SE v2.0.12, rRNA genes with Barrnap v0.9, and other non-coding RNAs were identified with Infernal v1.1.5 by comparison with the Rfam database (Li et al., 2023). Protein-coding genes were predicted using Prodigal v2.6.3 and GeneMarkS v4.3. Prophage regions and genomic islands were predicted using the corresponding mobile genetic element analysis tools, including Phigaro v2.4.0, IslandPath-DIMOB v1.0.0, and Islander v1.2, following the genome annotation workflow used in the original analysis (Milton and Cavanagh, 2023; Musik et al., 2021). Predicted protein sequences were searched against the NCBI NR (release 20230830), eggNOG (2020.06), KEGG (release 20230830), Swiss-Prot (release 202312), GO (release 20230830), TCDB (v20240820), Pfam (v36), CAZy (v12), and CARD (v3.2.9) databases using DIAMOND v0.8.35. Unless otherwise specified, annotations were retained when the *E*-value was ≤ 1e-5, sequence identity was ≥ 40%, and query coverage was ≥ 40%. Secondary metabolite biosynthetic gene clusters were predicted using antiSMASH v7.0.0 with the MIBiG database as the reference. Biosafety-related screening also considered VFDB (release 20240301), PHI (v5.0), and ResFinder v4.5.0 annotations. Based on these database annotations, candidate genes related to plant growth promotion, nutrient transformation, environmental adaptation, heavy metal tolerance, antibiotic resistance, and virulence-associated traits were screened. Functional gene annotations were interpreted as predicted genetic potential unless supported by phenotypic or physiological evidence.

#### 2.2.3 Determination of biofilm formation and rhizosphere colonization ability

Biofilm formation assay: Biofilm formation was determined with slight modifications based on previously reported methods (Shen, 2024). For qualitative observation, a 48-well plate assay was used. The strain was cultured in NB medium to the logarithmic phase, and the bacterial suspension was adjusted to OD600 = 1.0 with sterile medium. Each well received 1 mL MSgg medium and 10 μL bacterial suspension. After static incubation at 28°C for 16–24 h, the supernatant was carefully removed, the wells were gently rinsed with sterile water, and the morphology and wrinkled structure of the biofilm were observed under a stereomicroscope. For quantitative determination, a 6-well plate assay was used. Each well contained 10 mL MSgg medium and 100 μL bacterial suspension and was incubated under the same conditions. After incubation, biofilms formed on the well wall and liquid surface were collected, filtered through a 0.22 μm membrane, dried to constant weight, and quantified according to the weight difference of the membrane before and after filtration.

Rhizosphere colonization assay: Rhizosphere colonization was determined with slight modifications based on previously reported methods (Feng, 2020; Shen, 2024). Chinese cabbage seeds were surface-sterilized and cultured on MS solid medium for 5–6 days to obtain axenic seedlings. YNK-FB0053 was cultured and adjusted to OD600 = 0.5, and the roots of axenic seedlings were immersed in the bacterial suspension for 2 h. The seedlings were then transferred to a sterile culture system and incubated for 2 days. A portion of the root samples was fixed, dehydrated, dried, and sputter-coated with gold for scanning electron microscopy to observe bacterial attachment on the root surface. Another portion was used for plate counting. For plate counting, roots were fully eluted in sterile physiological saline, serially diluted, spread on NA plates, and incubated at 28°C for 24–48 h. Plates containing 30–300 colonies were selected, and the colonization density was calculated as follows: rhizosphere colonization density (CFU/g root fresh weight) = colony number on the plate × dilution factor × total volume of eluent/root fresh weight.

#### 2.2.4 Pot experiment and determination of related indicators

To investigate the effects of *Burkholderia gladioli* YNK-FB0053 on the growth and sulfur utilization efficiency of Chinese cabbage, a greenhouse pot experiment was conducted using a randomized block design. Plastic pots of identical size (22 cm upper diameter × 17.5 cm bottom diameter × 19.5 cm height) were each filled with 4.0 kg of air-dried, sieved, and homogenized soil. One Chinese cabbage seedling was transplanted into each pot. Two sulfur conditions were established: without sulfur fertilizer and with sulfur fertilizer. In the sulfur-fertilized treatments, elemental sulfur fertilizer containing 84% S was thoroughly mixed with the soil before transplanting at a rate equivalent to 50 mg S kg^–1^ dry soil. Each sulfur condition included five liquid treatments:

Without sulfur fertilizer: T1-sterile water, T2-nutrient broth medium, T3-24 h fermentation broth, T4-10-fold diluted fermentation broth, T5-100-fold diluted fermentation broth;

With sulfur fertilizer: T6-sulfur fertilizer + sterile water, T7-sulfur fertilizer + nutrient broth medium, T8-sulfur fertilizer + 24 h fermentation broth, T9-sulfur fertilizer + ten-fold diluted fermentation broth, T10-sulfur fertilizer + 100-fold diluted fermentation broth.

Each treatment included three biological replicates, with each replicate consisting of one pot containing one Chinese cabbage seedling. Each pot was regarded as an independent experimental unit. Seven days after transplantation, when the seedlings had adapted to the pot conditions, 100 mL of the corresponding treatment solution was applied to each pot by root drenching. The same treatment was repeated once after a 7-day interval. During the growth period, all pots were managed under the same greenhouse conditions and watered every 3–5 days.

At 50 days after transplantation, plant growth indices were determined, including chlorophyll content (SPAD value), plant height, root length, stem diameter, shoot dry weight, root dry weight, and leaf number. At harvest, shoots were cut at the soil surface and collected separately. To minimize root loss, the whole soil block was carefully loosened from each pot and gently immersed in water to soften the soil surrounding the root system. The roots were then slowly separated from the soil over a fine mesh sieve under a gentle water flow to retain fine lateral roots as much as possible. Residual soil particles were carefully removed, and visible non-root debris was discarded. After washing, the roots were blotted dry with absorbent paper before root length and biomass measurements. Shoots and roots were dried separately to constant weight for dry biomass determination.

For nutrient analysis, the aboveground plant samples collected at 50 days after transplantation were heat-treated for enzyme inactivation, oven-dried, ground, and sieved. The contents of nitrogen (N), phosphorus (P), and potassium (K) were determined according to the standard method for determination of nitrogen, phosphorus, and potassium in plants (NY/T 2017—2011), and sulfur (S) and iron (Fe) contents were measured according to the standard method for determination of total silicon, iron, and other elements in forest plants and forest litter (LY/T 1270—1999). Rhizosphere soil was collected at the same harvest stage, and pH, total nitrogen (TN), total phosphorus (TP), total potassium (TK), hydrolyzable nitrogen (AN), available potassium (AK), available phosphorus (AP), soil organic carbon (SOC), available sulfur (SO_4_^2–^), and total sulfur (TS) were determined according to Soil Agricultural Chemical Analysis Methods (Lu, 2000).

### 2.3 Data statistics and analysis

The experimental data were expressed as mean ± standard deviation (Mean ± SD). Each treatment contained three independent biological replicates, and each replicate consisted of one pot with one plant. Three biological replicates are commonly used in controlled greenhouse pot experiments for treatment-level evaluation, particularly when the experiment includes multiple treatments and all pots are managed under the same environmental conditions using a randomized block design. SPSS 26.0 software was used for one-way analysis of variance (ANOVA), and Duncan’s multiple comparison method was used for pairwise comparison between groups. A *P* < 0.05 was considered statistically significant. Origin 2022 software was used for graphing.

## 3 Results and analysis

### 3.1 Genome sequencing and basic characteristics

Whole-genome sequencing, assembly, and quality assessment of strain YNK-FB0053 were performed using a combined Illumina NovaSeq and PacBio Sequel strategy. Illumina sequencing generated 996,434,009 bp of clean bases, with clean Q20 and Q30 values of 97.82 and 90.65%, respectively. PacBio sequencing generated 548,213,285 bp of reads, with an average read length of 10,518.29 bp and a reads N50 of 10,547 bp. Based on the assembled genome size, the PacBio sequencing depth was 67.28 × . The read-mapping coverage of both Illumina and PacBio reads was 100%, and the K-mer-based genome coverage was 93.87%. CheckM analysis estimated genome completeness as 100%, with 1.68% contamination and 0% strain heterogeneity. BUSCO analysis showed 100% complete BUSCOs and 100% single-copy BUSCOs, with no duplicated, fragmented, or missing BUSCOs. These results indicate that the YNK-FB0053 genome assembly has high completeness and reliability.

**FIGURE 1:**
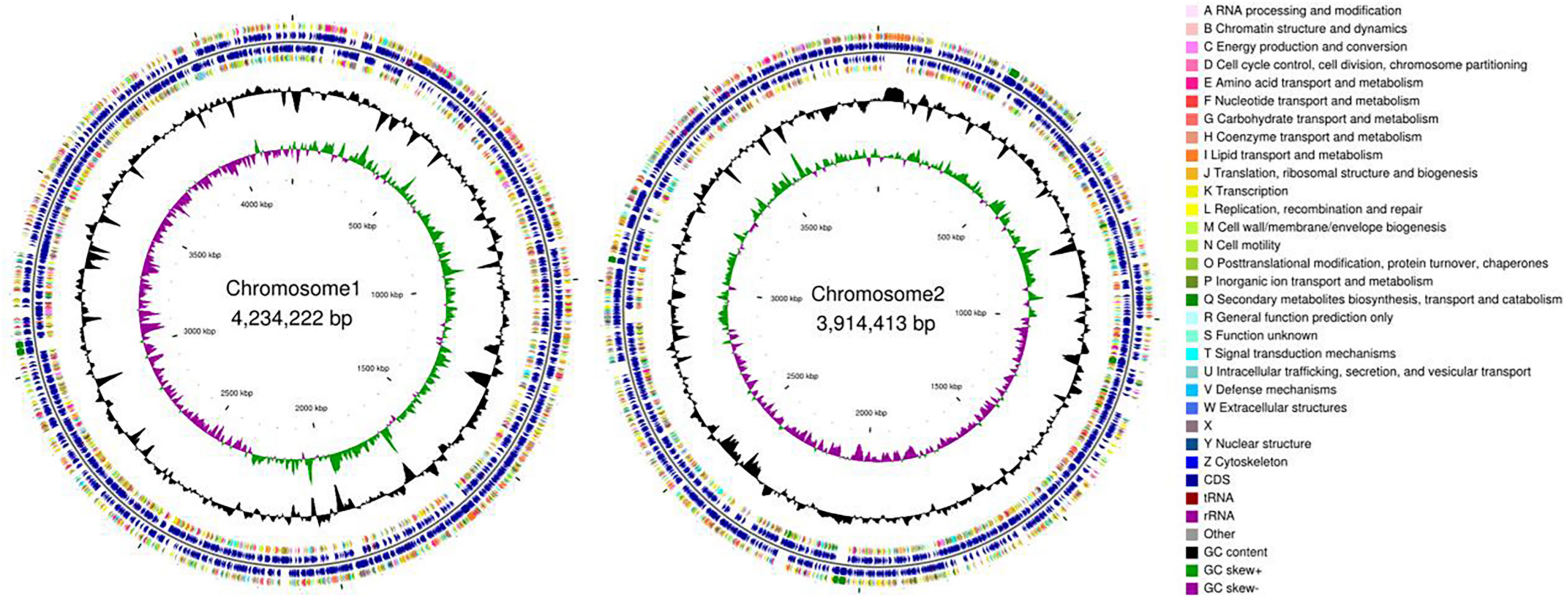
Circular genome map of strain YNK-FB0053. From the inside out, the concentric circles represent the following information in order: the first circle indicates the scale marker; the second circle shows the distribution of GC skew values; the third circle reflects the GC content variation; the fourth and seventh circles display the COG functional categories corresponding to each CDS; and the fifth and sixth circles mark the precise genomic locations of CDS, tRNA, and rRNA.

The final assembly consisted of two circular chromosomes of 4,234,222 and 3,914,413 bp, respectively, with a total genome size of 8,148,635 bp and a GC content of 68.07%. The assembly N50 was 4,234,222 bp, and no plasmid was detected. A total of 6,898 protein-coding genes were predicted, with a total coding length of 7,055,307 bp, a coding ratio of 86.58%, and an average coding gene length of 1,022.80 bp (Figure 1). The genome contained 65 tRNA genes representing 20 tRNA types, 15 rRNA genes (five copies each of 16S rRNA, 23S rRNA, and 5S rRNA), 31 housekeeping genes, and 56 sRNA genes. In addition, 148 tandem repeat sequences, 6 prophages, 20 genomic islands, 1,643 transmembrane protein-coding genes, and 95 secretory protein-coding genes were identified. The genome sequencing data of this strain have been submitted to GenBank under accession number JBQPHS000000000 (Table 1).

**TABLE 1:** Genomic characteristics of strain YNK-FB0053.

Characteristics	Value
Genome size (bp)	8,148,635
Chromosome1 size (bp)	4,234,222
Chromosome2 size (bp)	3,914,413
GC content (mol%)	68.07
Protein-coding genes (CDS)	6,898
tRNA	65
rRNA (5S, 16S, 23S)	15
House-keeping gene	31
sRNA	56
Tandem repeat	148
Prophage	6
Genomic islands	20
Transmembrane protein-coding genes	1,643
Secretory protein-coding genes	95
GenBank accession number	JBQPHS000000000
No. of plasmids	0
Gene total length (bp)	7,055,307
Average coding gene length (bp)	1,022.80
PacBio sequencing depth (x)	67.28
Illumina clean data (bp)	996,434,009
PacBio total bases (bp)	548,213,285
Reads mapping coverage (%)	Illumina 100; PacBio 100
K-mer-based genome coverage (%)	93.87
CheckM completeness/contamination (%)	100/1.68
BUSCO complete/single-copy (%)	100/100
Illumina sequencing depth ( × )	122.28
Overall sequencing depth ( × )	189.56
Assembly N50 (bp)	4,234,222
CheckM strain heterogeneity (%)	0
BUSCO duplicated/fragmented/missing (%)	0/0/0

### 3.2 Genome functional annotation

**FIGURE 2:**
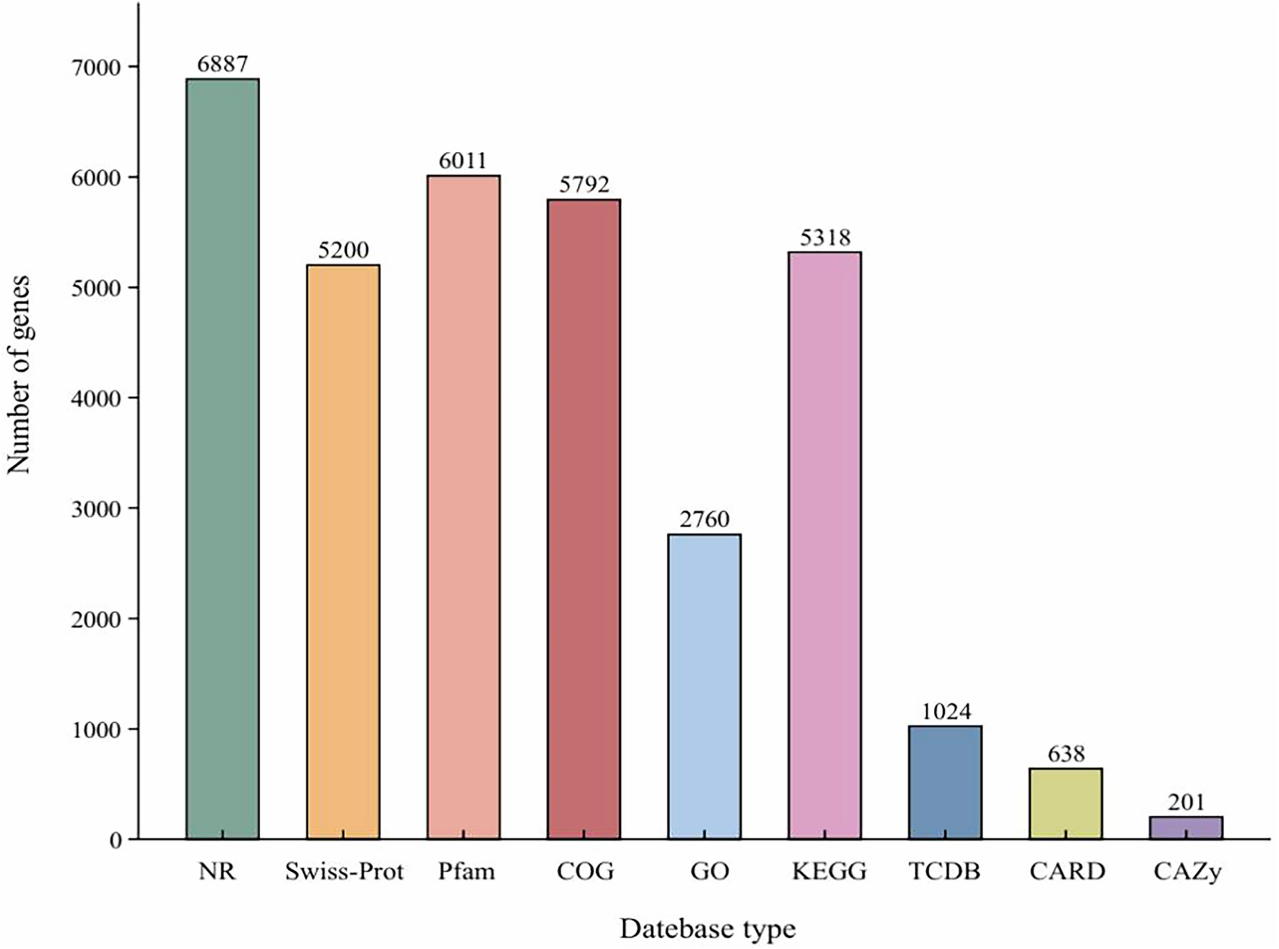
Distribution of functional annotations for strain YNK-FB0053 genes across different databases.

The protein sequences of the predicted genes were aligned with databases such as NR, GO, and KEGG using Diamond (*E* ≤ 1e-5, identity ≥ 40%, coverage ≥ 40%). There were differences in the number and proportion of gene annotations among the databases (Figure 2): 6,887 genes were annotated in the NR database (99.84%), 5,200 in Swiss-Prot (75.38%), 6,011 in Pfam (87.14%), 5,792 in COG (83.97%), 5,318 in KEGG (77.09%), 2,760 in GO (40.01%), 1,024 in TCDB (14.84%), and 201 in CAZy (2.91%). The CARD search produced 638 raw database hits, which were further filtered to 72 non-redundant antibiotic resistance-related genes for biological interpretation. Among these, the NR database had the highest annotation rate, while the CAZy database had the lowest.

### 3.3 COG database annotation

**FIGURE 3:**
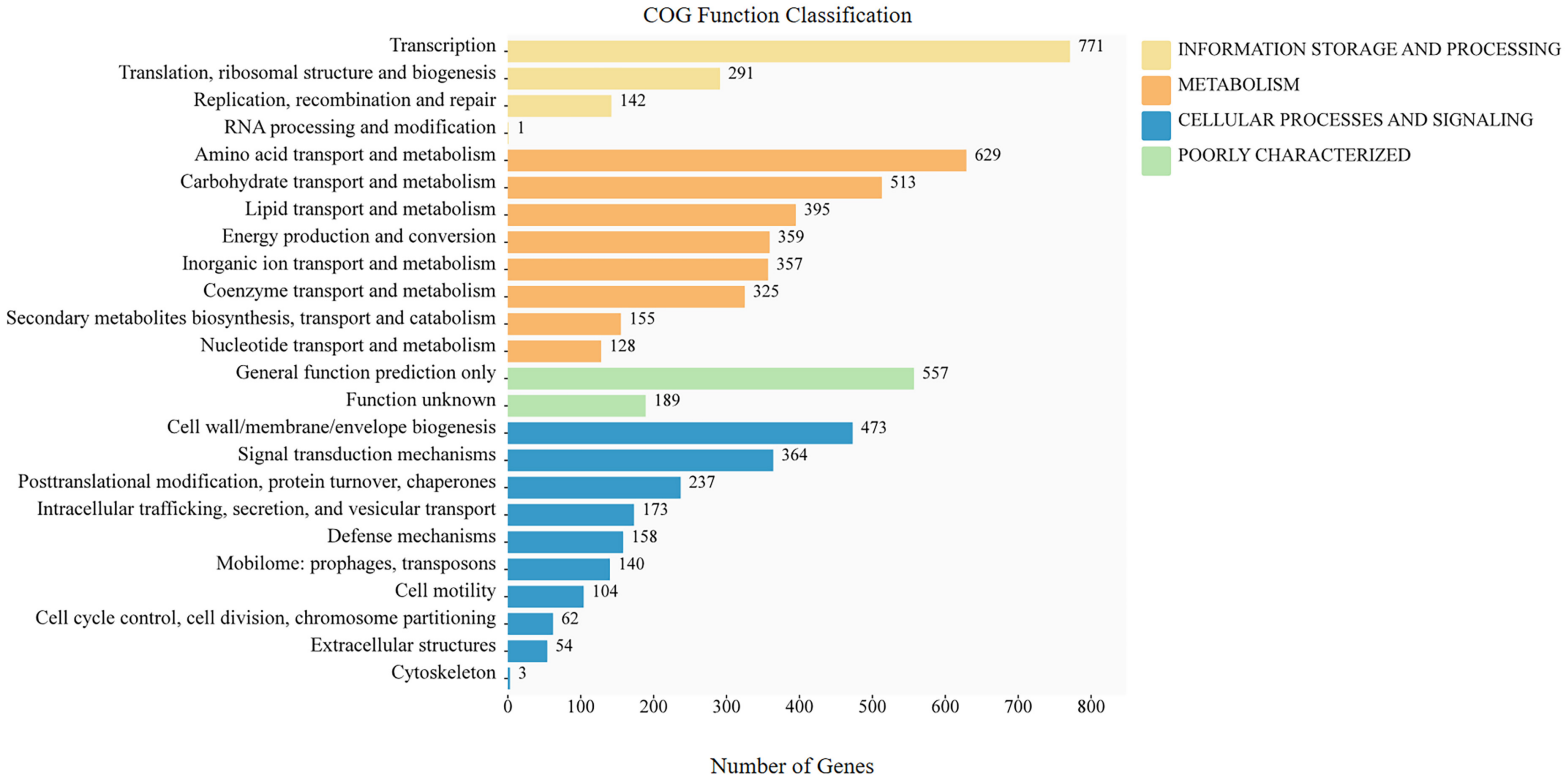
Functional annotation results of the strain YNK-FB0053 genome based on the COG database.

A total of 5,792 protein-coding genes were annotated in the COG database, which were classified into 4 major categories and 24 subcategories (Figure 3). Among them, there were 2,861 metabolic genes (49.40%), 746 genes related to cellular processes and signaling (12.88%), 1,205 genes involved in information storage and processing (20.80%), and 1,768 genes with unclear annotation characteristics (30.52%). These annotations suggest that the strain has broad predicted metabolic and environmental adaptation potential, but the specific biological activities require phenotypic confirmation.

Among the 24 subcategories, the number of transcription-related genes was the largest (771, 13.31%), followed by amino acid transport and metabolism (629, 10.86%). Genes related to general function prediction (557, 9.62%), carbohydrate transport and metabolism (513, 8.86%), and cell wall/membrane/envelope biogenesis (473, 8.17%) also accounted for relatively high proportions. These results demonstrate that the strain has significant potential in functional expression, nutrient competition, and environmental adaptation.

### 3.4 GO database annotation

**FIGURE 4:**
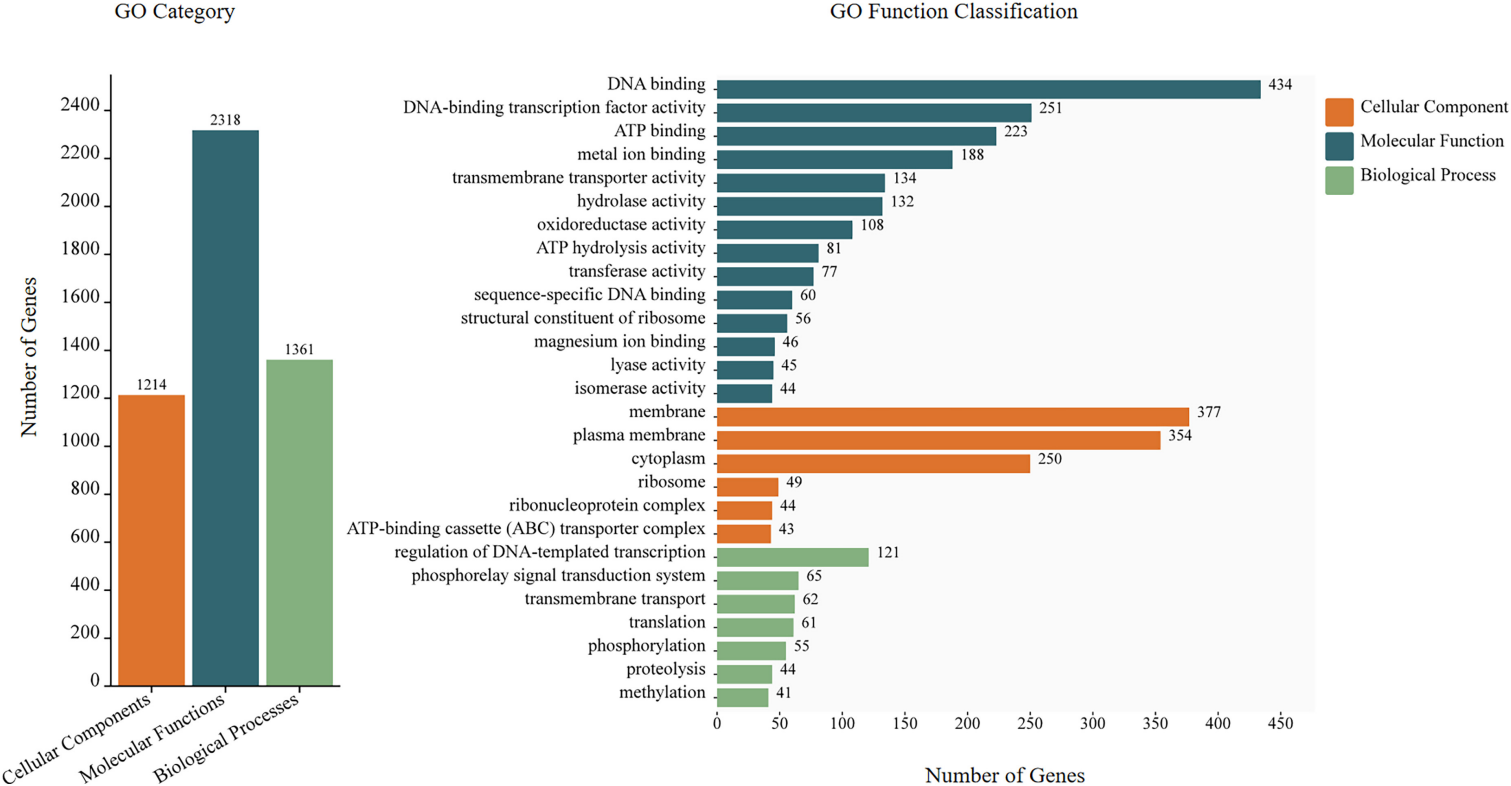
Functional annotation results of the strain YNK-FB0053 genome based on the GO database.

A total of 2,760 genes were annotated in the GO database, which categorizes proteins into three aspects: biological process, cellular component, and molecular function (Figure 4). There are 1,351 branches in total, with 484, 66, and 801 branches in biological process, cellular component, and molecular function, respectively.

In the biological process category, 1,898 genes were annotated, and the most abundant genes were associated with the regulation of DNA-templated transcription, phosphorelay signal transduction system, transmembrane transport, and translation, with 121, 65, 62, and 61 genes, respectively. In the cellular component category, 1,370 genes were annotated, among which genes related to membrane, plasma membrane, and cytoplasm showed the highest relevance, with 377, 354, and 250 genes, respectively. In the molecular function category, 4,051 genes were annotated, and the most prominent genes were involved in DNA binding, DNA-binding transcription factor activity, and ATP binding, with 434, 251, and 223 genes, respectively.

### 3.5 KEGG database annotation

**FIGURE 5:**
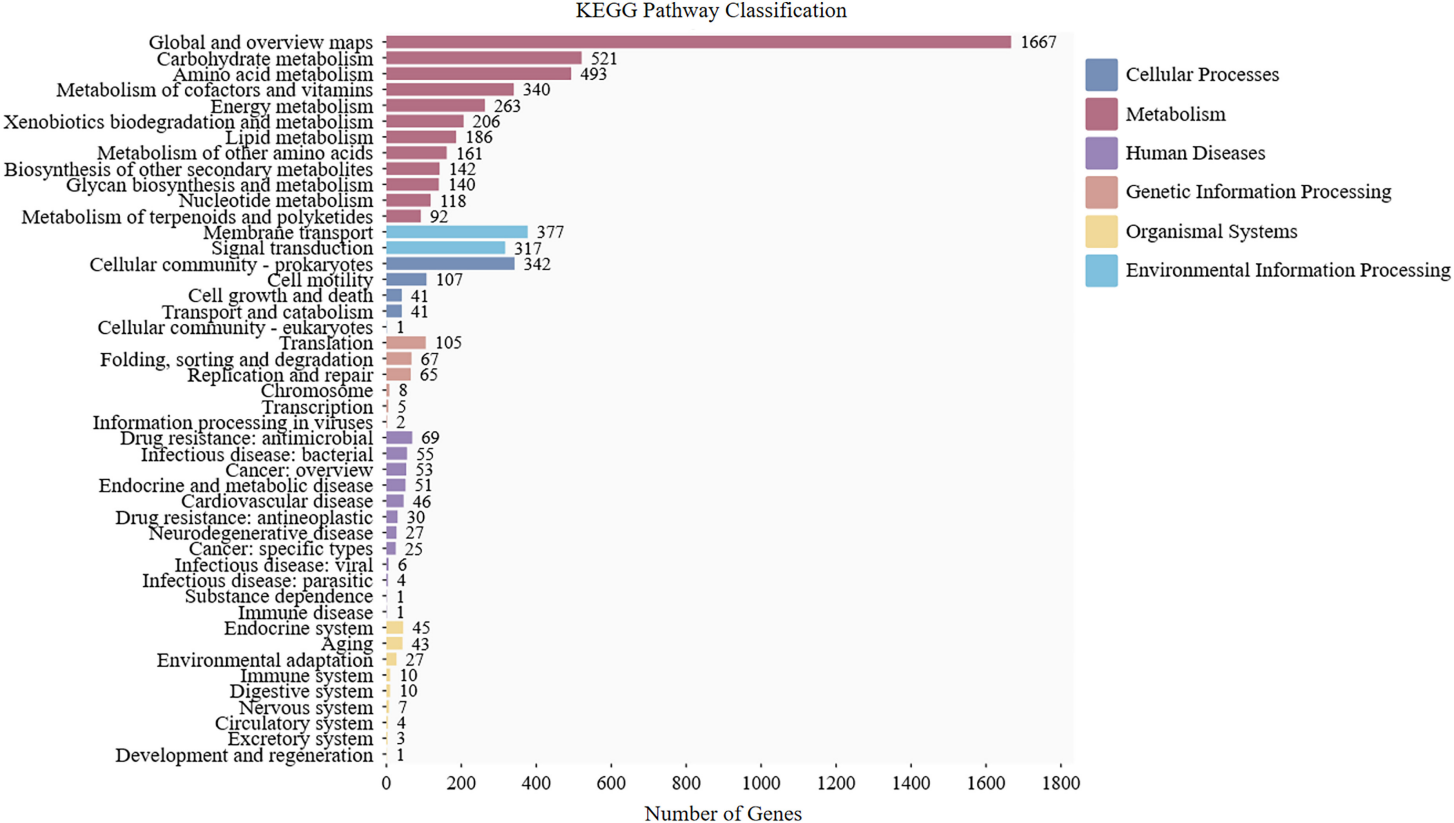
Functional annotation results of the strain YNK-FB0053 genome based on the KEGG database.

A total of 6,325 genes were annotated in the KEGG database, which includes 6 major categories (Metabolism, Genetic Information Processing, Environmental Information Processing, Human Diseases, Organismal Systems, and Cellular Processes) and 44 subcategories (Figure 5). Even with some genes assigned to multiple categories, Metabolism was the most abundant category with 4,329 genes (68.44%).

Among the 44 subcategories, the annotation of “Global and overview maps” was particularly prominent (1,667 genes, 23.36%). Beyond this, “Carbohydrate metabolism” had the highest number of genes (521, 8.24%), followed by “Amino acid metabolism” (493, 7.79%). Additionally, relatively high annotations were observed for “Membrane transport” (377, 5.96%), “Cellular community-prokaryotes” (342, 5.41%), “Metabolism of cofactors and vitamins” (340, 5.38%), and “Signal transduction” (317, 5.01%).

### 3.6 CAZyme database annotation

**FIGURE 6:**
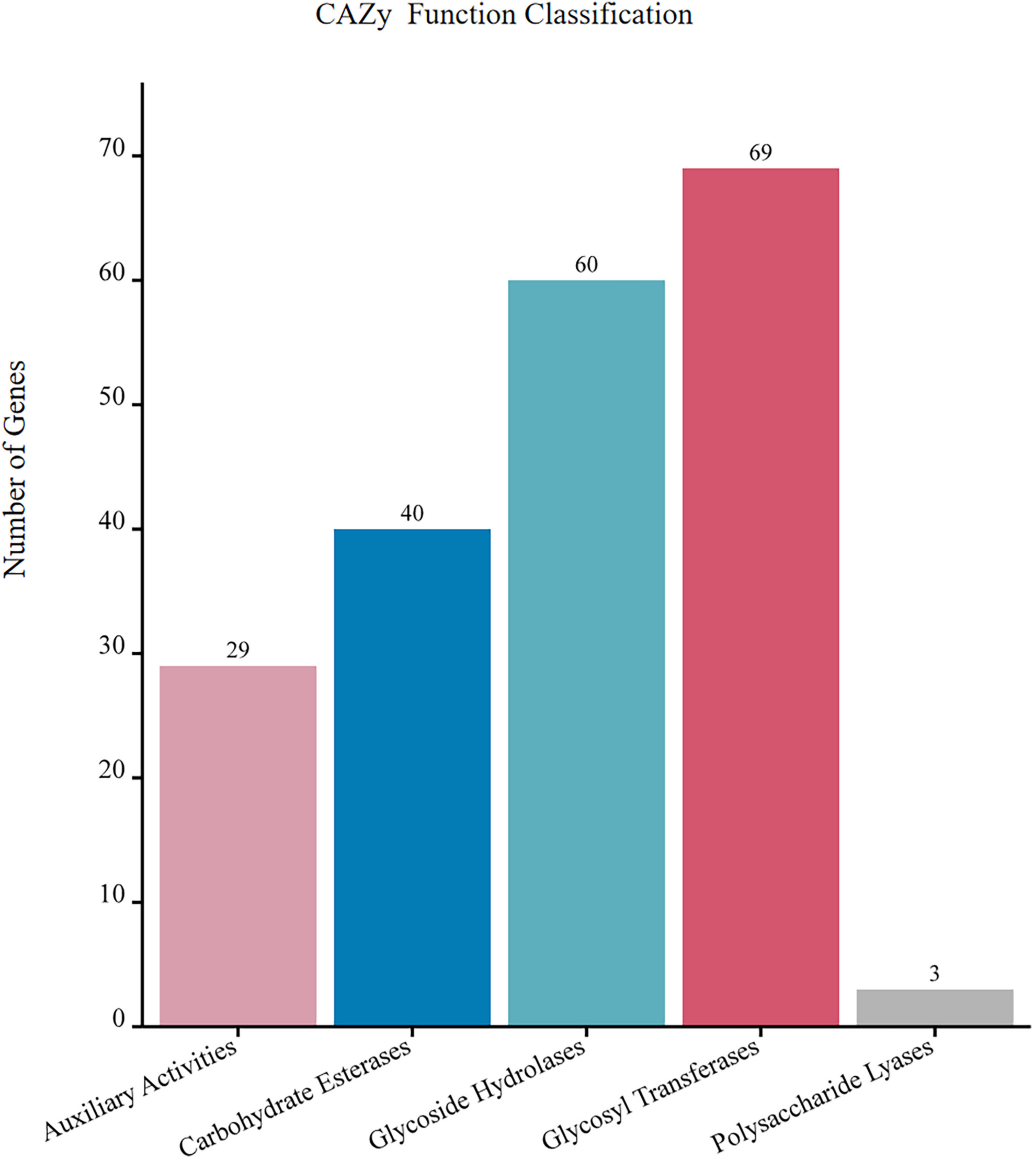
Functional annotation results of the strain YNK-FB0053 genome based on the CAZy database.

Genome sequence alignment with the CAZy database showed that the genome of strain *Burkholderia gladioli* YNK-FB0053 contains 201 genes whose encoded protein domains belong to the CAZy family (Figure 6). These include 60 proteins from 40 glycoside hydrolase (GHs) families, 69 proteins from 14 glycosyltransferase (GTs) families, 40 proteins from 8 carbohydrate esterase (CEs) families, 29 proteins from 8 auxiliary activity (AAs) families, and 3 proteins from polysaccharide lyase (PLs) families.

Among these, families associated with chitin degradation, starch hydrolysis, and cellulose degradation (e.g., GH3, GH15, GH18, GH19, GH23, and GH39) were identified. Meanwhile, the genome of strain YNK-FB0053 harbors a large number of genes encoding endo-beta-1,4-glucanase and beta-glucosidase, which are involved in decomposing and absorbing carbohydrates and proteins from plant disease residues in soil.

### 3.7 CARD database annotation

**FIGURE 7:**
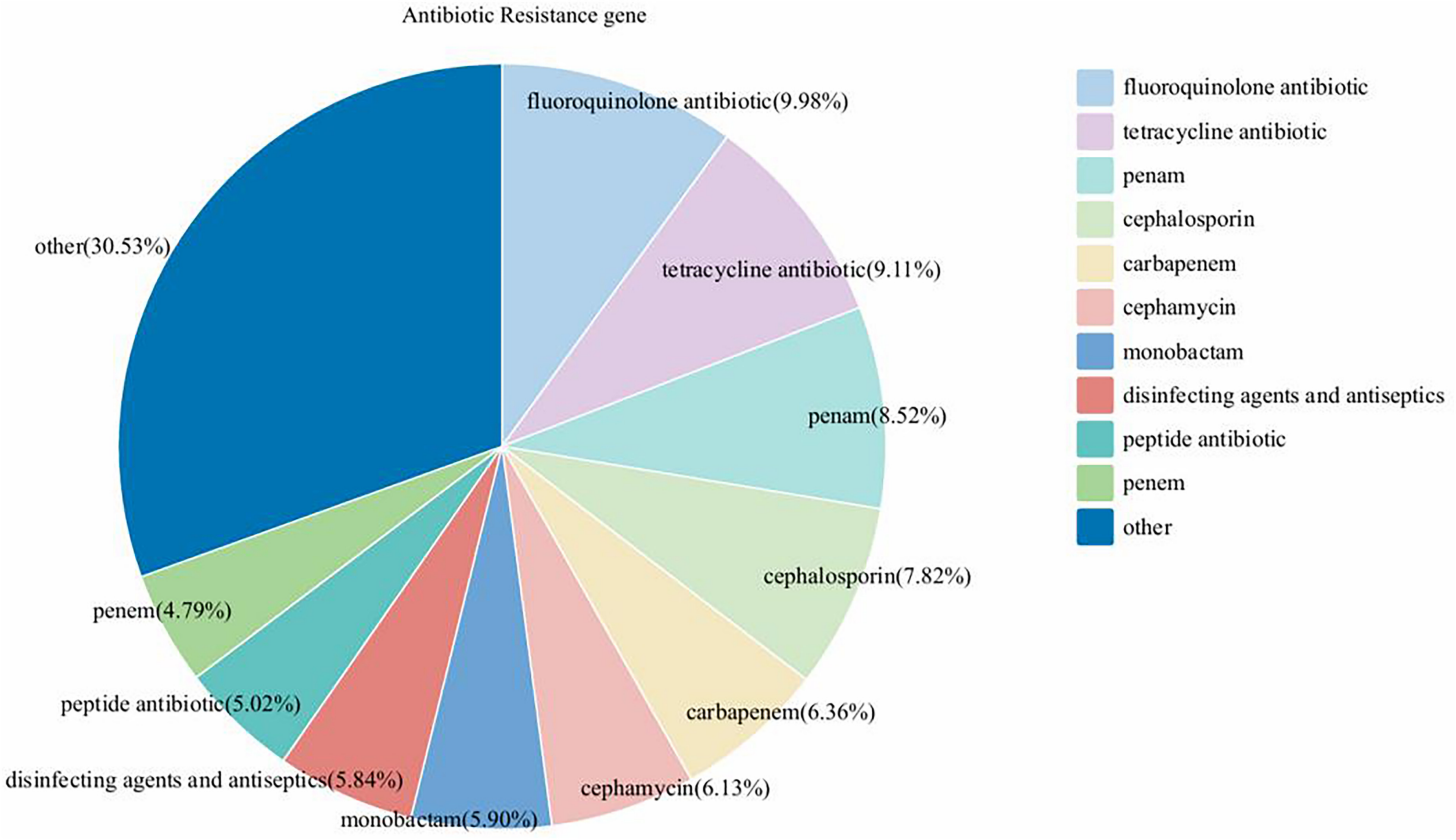
Functional annotation results of the strain YNK-FB0053 genome based on the CARD database.

Genome alignment of strain YNK-FB0053 against the CARD database produced 638 resistance-related assignments. After removing redundant and overlapping annotations, 72 non-redundant antibiotic resistance-related genes were retained for biological interpretation. At the antibiotic class level, the most frequently represented CARD assignments were associated with fluoroquinolone antibiotics (171 assignments, 9.98%), tetracycline antibiotics (156 assignments, 9.11%), penam antibiotics (146 assignments, 8.52%), and cephalosporins (134 assignments, 7.82%) (Figure 7).

In terms of predicted resistance mechanisms, antibiotic efflux was the most abundant category, with 389 assignments, followed by antibiotic target alteration with 132 assignments, reduced membrane permeability with 56 assignments, and antibiotic inactivation with 34 assignments. Because individual CARD entries may be associated with multiple antibiotic classes or resistance mechanisms, these values represent database-derived assignment counts rather than independent gene numbers. Therefore, the CARD results indicate potential resistance-related genetic determinants in the genome of YNK-FB0053, and phenotypic antibiotic resistance should be further evaluated through antibiotic susceptibility testing, especially before field-scale agricultural application.

### 3.8 Annotation of secondary metabolite biosynthetic gene clusters

*Burkholderia* species typically possess large and structurally complex genomes enriched in biosynthetic gene clusters (BGCs) responsible for secondary metabolite production. In this study, the genome of strain YNK-FB0053 was systematically analyzed using antiSMASH v7.0.0, with the MIBiG database as a reference. A stringent cluster detection strategy was applied to predict its potential for secondary metabolite biosynthesis.

The results identified a total of 19 biosynthetic gene clusters in the genome of strain YNK-FB0053 (Supplementary Table S1), including 3 terpene clusters, 3 RiPP-like clusters, 3 type I polyketide synthase (T1PKS) clusters, 1 homoserine lactone (hserlactone) cluster, 1 redox-cofactor cluster, 1 phosphonate cluster, and 7 nonribosomal peptide synthetase (NRPS) clusters.

Among all predicted clusters, only four showed 100% similarity to known biosynthetic gene clusters, corresponding to enacyloxin IIa, rhizomide A/B/C, sulfazecin, and icosalide A/B, with their cluster architectures illustrated in Supplementary Figure S1. The remaining 15 clusters exhibited < 85% similarity to previously reported clusters, among which five clusters showed no detectable homologous sequences, suggesting their potential to encode novel secondary metabolites.

In addition, homoserine lactone (hserlactone)-related structures were predicted in some clusters. These compounds typically function as signaling molecules in quorum sensing systems, indicating that strain YNK-FB0053 may possess quorum sensing regulatory capabilities. This feature is consistent with the characteristic physiological traits of the genus *Burkholderia*.

### 3.9 Genes involved in sulfur oxidation pathways

Sulfur oxidation is a central functional focus of this study. Whole-genome annotation revealed that strain YNK-FB0053 carries multiple sulfur metabolism-related candidate genes associated with reduced sulfur oxidation, thiosulfate-related transformation, sulfate/thiosulfate transport, assimilatory sulfate reduction, and organic sulfur utilization, providing a genetic basis for its sulfur-related functional potential.

The genome contains *sqr*, which encodes sulfide:quinone oxidoreductase and is predicted to participate in the oxidation of reduced sulfide, with electrons transferred to the respiratory chain. Sox-system-related genes, including *soxA*, *soxB*, *soxC*, *soxD*, and *soxG*, were also identified. These genes are commonly associated with the oxidation of thiosulfate and other reduced sulfur compounds in sulfur-oxidizing microorganisms. Among them, *soxB* is generally involved in the hydrolysis of sulfur-bound intermediates, whereas *soxC* and *soxD* are associated with further oxidation of sulfur intermediates. However, a complete canonical *soxXYZABCD* cluster was not annotated in YNK-FB0053, and *tsdA*, which encodes thiosulfate dehydrogenase in some alternative thiosulfate oxidation pathways, was not detected. Therefore, the sulfur-oxidizing potential of YNK-FB0053 is more appropriately interpreted as being associated with an *sqr*-linked sulfide oxidation route and Sox-related sulfur oxidation components, rather than a complete canonical Sox pathway or a *tsdA*-dependent pathway. Together with the previously reported sulfate production phenotype, these genes provide a genomic explanation for the sulfur-oxidizing potential of this strain, although the catalytic efficiency, substrate preference, and metabolic flux require further physiological and biochemical validation.

In addition, the genome contains the *cysPUWA* sulfate/thiosulfate transport system, including *cysP*, *cysU*, *cysW*, and *cysA*, suggesting the potential for sulfate and thiosulfate uptake. Assimilatory sulfate reduction-related genes, including *cysN*, *cysD*, *cysC*, *cysH*, and *cysI*, were also detected. In this pathway, sulfate can be activated to adenosine 5′-phosphosulfate (APS) by sulfate adenylyltransferase encoded by *cysN* and *cysD*, further converted to 3′-phosphoadenosine-5′-phosphosulfate (PAPS) by adenylylsulfate kinase encoded by *cysC*, reduced to sulfite by phosphoadenosine phosphosulfate reductase encoded by *cysH*, and then further reduced by sulfite reductase components such as *cysI*. These genes suggest that YNK-FB0053 has the genetic potential to incorporate sulfate-derived sulfur into cellular sulfur metabolism and sulfur-containing compounds. This assimilatory pathway may also contribute to the formation of reduced sulfur metabolites during bacterial sulfur metabolism, but the actual production level and physiological role of these metabolites require further targeted detection.

The presence of *ssuABC*, *ssuD*, *ssuE*, *tauABC*, and *sfnG* further suggests that YNK-FB0053 may utilize diverse organic sulfur sources, such as sulfonates, taurine, and methylated sulfur compounds. This potential capacity for organic sulfur utilization may broaden the sulfur-source spectrum of the strain in the rhizosphere and contribute to soil sulfur turnover.

Taken together, YNK-FB0053 carries sulfur metabolism-related genes corresponding to reduced sulfide oxidation, Sox-related sulfur transformation, sulfate/thiosulfate transport, assimilatory sulfate reduction, and organic sulfur utilization. This sulfur-related genetic architecture, together with the previously reported sulfate production phenotype, supports the potential role of this strain in promoting sulfur transformation and increasing plant-available sulfur under rhizosphere conditions. In addition, sulfur transformation may contribute to changes in the rhizosphere microenvironment and thereby indirectly influence the availability of other nutrients such as phosphorus. Nevertheless, the specific sulfur substrates, intermediate metabolites, enzyme activities, and metabolic fluxes should be further confirmed through targeted substrate-utilization assays, sulfur metabolite profiling, and enzymatic activity measurements. Sulfur metabolism-related genes identified in this study are listed in Supplementary Table S2.

### 3.10 Plant growth-promoting related genes

#### 3.10.1 Nutrient transformation capacity

##### 3.10.1.1 Phosphate solubilization

Phosphorus is an essential nutrient for plant growth; however, a large proportion of soil phosphorus exists in insoluble forms, limiting its bioavailability. Genome annotation revealed that strain YNK-FB0053 harbors multiple genes predicted to be associated with phosphorus cycling, including inorganic phosphate mobilization, organic phosphorus mineralization, phosphate transport, and phosphate starvation response regulation.

For inorganic phosphate mobilization, multiple *gcd* and *gdh* genes were identified, along with genes involved in pyrroloquinoline quinone (PQQ) biosynthesis, including *pqqB*, *pqqC*, *pqqD*, and *pqqE*. These genes suggest the presence of a PQQ-dependent glucose dehydrogenase-related system, which may contribute to organic acid production and thus potentially facilitate the mobilization of insoluble phosphate. In addition, the presence of *ppa* and *ppx–gppA* suggests potential involvement in the conversion of pyrophosphate and polyphosphate into inorganic phosphate. The identification of *glpQ* further suggests a potential capacity to utilize glycerophospholipid-derived organic phosphorus compounds, thereby contributing to organic phosphorus mineralization.

In terms of phosphorus uptake and regulation, strain YNK-FB0053 harbors genes encoding the high-affinity phosphate transport system *pstSCAB*, as well as key components of the Pho regulon, including *phoB*, *phoR*, *phoU*, and *phoH*. These genes are predicted to participate in phosphate acquisition and phosphate-starvation response regulation under phosphate-limited conditions. Additionally, the genome encodes TC.PIT transporters and genes such as *ugpABCE* and *phnF*, suggesting potential involvement in the uptake or regulation of multiple phosphorus sources, including inorganic phosphate, glycerol-3-phosphate, and phosphonate-related compounds. Relevant genes are listed in Supplementary Table S3.

##### 3.10.1.2 Nitrogen cycling

Cultivation assays showed that strain YNK-FB0053 could grow on Ashby nitrogen-free medium, suggesting its possible adaptation to low-nitrogen conditions. However, the canonical nitrogenase structural genes *nifH*, *nifD*, and *nifK* were not detected in the current genome annotation; therefore, nitrogen-fixing ability cannot be inferred solely from growth on Ashby medium or from the presence of individual auxiliary genes. Genome analysis indicated that this strain harbors multiple genes related to nitrogen transformation and assimilation, mainly involving nitrate/nitrite transport and reduction, ammonium assimilation, and metabolism of cyanate-containing nitrogen compounds.

For nitrogen redox and transport processes, multiple *narK* genes encoding nitrate/nitrite transporters were identified, suggesting potential involvement in nitrate uptake and associated metabolic processes. The presence of *nirB*, encoding the large subunit of NADH-dependent nitrite reductase, indicates a predicted capacity to reduce nitrite to ammonium.

Regarding nitrogen assimilation, key genes including *glnA*, *gdhA*, and *glnB* were identified, forming pathways related to ammonium assimilation and nitrogen-status regulation. Specifically, *glnA* encodes glutamine synthetase, a key enzyme for incorporating ammonium into amino acid biosynthesis, while *glnB* encodes the PII protein, which plays a central role in sensing cellular nitrogen status and regulating nitrogen metabolism. In addition, *cynS* and *cynT* suggest the ability to convert cyanate into ammonium, enabling the utilization of alternative nitrogen sources under nitrogen-limited conditions.

Notably, the *iscU* gene was also detected. However, *iscU* encodes an iron-sulfur cluster assembly protein, which mainly participates in iron-sulfur cluster biosynthesis and maturation of multiple enzyme proteins. It may support the structural and functional stability of some nitrogen metabolism-related enzymes, but it is not a nitrogenase structural gene and cannot be used as direct evidence for nitrogen fixation. Overall, the genomic evidence supports relatively complete inorganic nitrogen transformation and assimilation potential in YNK-FB0053, whereas nitrogen-fixing ability still requires further verification using nif gene detection, acetylene reduction assays, or ^15^N isotope tracing. Relevant genes are listed in Supplementary Table S4.

##### 3.10.1.3 Zinc solubilization

Zinc is an essential micronutrient for both plants and microorganisms, functioning as a cofactor in numerous enzymatic reactions. However, zinc in soil is often present in insoluble forms, resulting in low bioavailability. Microorganisms can influence zinc availability by regulating its solubilization, uptake, and efflux.

Genome annotation showed that strain YNK-FB0053 harbors multiple candidate genes associated with zinc homeostasis regulation and transport. The presence of *zur*, encoding a Fur-family transcriptional regulator, suggests its possible involvement in regulating intracellular zinc uptake and efflux. Multiple copies of *zntA*, encoding a Zn^2+^/Cd^2+^-exporting ATPase, and *zntB*, encoding a zinc transporter, were also detected, suggesting that this strain may maintain intracellular zinc homeostasis under high-zinc conditions. In addition, genes associated with the *tonB*–*exbB*–*exbD* system may provide energy for outer membrane transport processes and participate in metal ion uptake. Together, these gene sets indicate that YNK-FB0053 has the genetic potential to regulate zinc uptake, transport, and homeostasis; however, its zinc-solubilizing ability still requires phenotypic validation. The related genes are listed in Supplementary Table S5.

#### 3.10.2 Genes associated with siderophore production and Indole-3-Acetic acid biosynthesis in YNK-FB0053

##### 3.10.2.1 Siderophore production

Iron is an essential transition metal ion for living organisms and is indispensable for microbial growth. Siderophores exhibit high affinity for Fe^3+^ and can chelate insoluble iron, converting it into soluble forms available for plant uptake. In addition, siderophore production enables microorganisms to compete with pathogens for Fe^3+^, thereby indirectly suppressing pathogen proliferation.

Whole-genome annotation revealed that strain YNK-FB0053 carries genes predicted to be involved in iron acquisition and siderophore-related processes, indicating a potential role in rhizosphere iron mobilization and improvement of iron availability. The genome encodes *entS*, a major facilitator superfamily transporter associated with siderophore export, and the *afuABC* high-affinity Fe^3+^ transport system, which may contribute to extracellular iron acquisition.

At the regulatory level, multiple *furB* genes may be involved in sensing intracellular iron levels and regulating iron transport-related genes. High-affinity iron transport proteins such as *efeU* and *ftrA*, together with AraC-type transcriptional activators, may enhance iron acquisition under iron-limited conditions. Combined with previous CAS phenotypic assay results, these gene annotations provide a genetic explanation for the siderophore-producing ability of YNK-FB0053; however, the specific siderophore types and production levels require further chemical identification. Detailed gene information is provided in Supplementary Table S6.

##### 3.10.2.2 Indole-3-acetic acid (IAA) production

Genome annotation indicated that strain YNK-FB0053 harbors genes involved in tryptophan biosynthesis and tryptophan-dependent indole-3-acetic acid (IAA) biosynthesis, providing a predicted molecular basis for phytohormone production. A complete tryptophan biosynthesis pathway encoded by *trpABCDEFG* was identified. Because tryptophan is a major precursor for microbial IAA synthesis, these genes suggest potential for IAA biosynthesis, although targeted IAA quantification is required for confirmation.

Furthermore, multiple genes encoding aldehyde dehydrogenases (ALDH) were identified, which may oxidize indole-3-acetaldehyde into IAA. Multiple copies of *amiE*, encoding amidase, were also detected, potentially enabling the conversion of indole-3-acetamide (IAM) into IAA. These findings suggest that strain YNK-FB0053 may possess multiple predicted IAA biosynthetic routes. Genes involved in IAA biosynthesis are listed in Supplementary Table S7.

#### 3.10.3 Degradation of phenolic compounds

In agricultural systems, continuous cropping may lead to the accumulation of phenolic and other aromatic compounds derived from plant root exudates and crop residues in the rhizosphere, which can negatively affect plant growth and microbial activity. Microbial degradation of aromatic compounds represents a potential biological mechanism for regulating the accumulation of these substances.

Genome analysis revealed that strain YNK-FB0053 harbors multiple genes predicted to be involved in aromatic compound degradation. In the benzoate degradation pathway, genes including *benA*, *benB*, *benC*, and *benD* were identified, suggesting the presence of a benzoate 1,2-dioxygenase-related system that may participate in the conversion of benzoate into catechol. In addition, the identification of the transporter gene *benE* and multiple copies of the regulatory gene *benM* suggests potential roles in substrate uptake and pathway regulation.

During the aromatic ring-cleavage stage, the strain carries *catA*, *catB*, *catC*, and *catE*, suggesting potential involvement in catechol degradation through ring-cleavage pathways. Furthermore, a *pca* gene cluster, including *pcaG*, *pcaH*, *pcaB*, *pcaC*, *pcaD*, *pcaI*, *pcaJ*, and *pcaF*, was identified, indicating a predicted capacity to metabolize compounds such as *p*-hydroxybenzoate and protocatechuate through the β-ketoadipate pathway and connect aromatic compound degradation with central metabolism.

Additionally, the presence of the transporter gene *pcaK* and regulatory genes *pcaR* and *pcaQ* suggests possible involvement in substrate uptake and inducible regulation of degradation-related genes. The genome also contains a relatively complete *paa* gene cluster, including *paaA–paaG*, *paaK*, and *paaI*, suggesting potential for the degradation of phenylacetic acid–type aromatic compounds. Relevant genes are listed in Supplementary Table S8.

#### 3.10.4 Rhizosphere colonization ability

Stable colonization of plant roots by plant growth-promoting microorganisms is a prerequisite for the sustained expression of their biological functions. Genome analysis of strain YNK-FB0053 revealed a relatively complete set of genes associated with chemotaxis sensing and motility regulation, suggesting a predicted molecular basis for directional movement toward the rhizosphere.

In terms of chemotaxis, the strain harbors multiple methyl-accepting chemotaxis protein (MCP)-related genes, including *tsr* and *mcp*, as well as energy- and oxygen-sensing receptor genes such as *aer*. Moreover, core components of the Che signal transduction system, including *cheA*, *cheW*, *cheY*, *cheB*, *cheR*, *cheZ*, and *cheV*, were identified in the genome. These genes are predicted to support the perception of environmental chemical gradients, signal integration, and regulation of motility responses, which may facilitate bacterial movement toward rhizosphere-associated chemical cues. Relevant chemotaxis-related genes are listed in Supplementary Table S9.

Regarding motility structures, strain YNK-FB0053 contains genes associated with flagellar assembly and regulation, including the master regulatory genes *flhDC*, the sigma factor gene *fliA*, structural genes involved in flagellar assembly (*fliC*, *fliD*, *fliE–fliK*, and *fliF–fliN*), and motor-associated genes (*motA* and *motB*). In addition, the presence of *flg* gene clusters suggests the potential for flagellar apparatus assembly, which may contribute to autonomous movement, surface attachment, and biofilm formation. Relevant genes are listed in Supplementary Table S10.

The chemotaxis system and flagellar motility system are functionally connected. Signal transduction mediated by the CheY protein can regulate flagellar motor switch proteins such as FliG, FliM, and FliN, allowing bacterial cells to adjust their movement direction and pattern in response to environmental cues. Furthermore, genes involved in extracellular polysaccharide (EPS) biosynthesis may contribute to bacterial adhesion to root surfaces, microbial aggregation, and stable biofilm formation. Collectively, these genomic features suggest that strain YNK-FB0053 has strong rhizosphere colonization potential. This prediction is further supported by the biofilm formation and root colonization assays described below. Genes related to EPS biosynthesis are listed in Supplementary Table S11.

#### 3.10.5 Heavy metal stress resistance and antibiotic resistance gene analysis

Strain YNK-FB0053 harbors multiple predicted genes associated with metal and metalloid tolerance, resistance regulation, and transmembrane efflux, involving Cu, Zn, Cd, Cr, As, and other stress-related elements. These genetic features suggest a potential basis for maintaining cellular metal homeostasis under metal stress. The genome contains a predicted *cusRS–cusCFBA* copper/silver resistance module, which may be involved in copper/silver sensing and efflux. In addition, P-type ATPase genes such as *copA* and *copB*, together with the copper-binding gene *copC*, may contribute to copper ion regulation and detoxification.

The presence of the CzcABC heavy metal efflux system, along with the accessory transporter gene *czcD*, suggests a potential capacity for the efflux of Zn^2+^, Cd^2+^, and Co^2+^ ions. Genes such as *chrA* and *chrR* are predicted to participate in chromate transport and Cr(VI) reduction, respectively, thereby potentially reducing chromium toxicity. Furthermore, the classical *arsRBC* resistance module suggests potential adaptation to arsenic stress. Overall, these predicted metal/metalloid resistance and efflux-related genes may contribute to the environmental adaptability of YNK-FB0053 under metal-stress conditions. However, their actual functional contribution requires further validation through metal-tolerance assays and gene-expression analyses. Detailed genes related to metal and metalloid stress are listed in Supplementary Tables S12–S15.

In addition, genome-wide annotation identified 72 non-redundant antibiotic resistance-related genes in strain YNK-FB0053, covering resistance-associated determinants for fluoroquinolones, tetracyclines, beta-lactams, and other antibiotic classes. These predicted resistance genes involve mechanisms such as antibiotic efflux, target modification, and reduced membrane permeability. Because *Burkholderia* species include opportunistic pathogens, these results highlight the need for further biosafety evaluation before agricultural application. Detailed information on these genes is provided in Supplementary Table S16.

### 3.11 Verification of rhizosphere colonization characteristics and plant growth–promoting effects of strain YNK-FB0053

Based on the genome functional annotation, YNK-FB0053 harbors candidate genes associated with chemotaxis and flagellar assembly, sulfur oxidation, inorganic nitrogen transformation and assimilation, phosphorus transformation, siderophore biosynthesis, and IAA biosynthesis. These genes not only provide a genetic basis for rhizosphere colonization but also suggest the functional potential of this strain to improve soil nutrient availability and promote plant nutrient uptake.

To systematically validate its actual colonization ability and plant growth-promoting effects, this study further conducted biofilm formation and rhizosphere colonization assays, pot experiments to evaluate agronomic traits and nutrient uptake, soil physicochemical property analyses, and correlation and principal component analyses to explore its possible mechanisms of action.

#### 3.11.1 Biofilm formation and rhizosphere colonization capacity

Genome annotation of strain YNK-FB0053 revealed the presence of genes related to chemotaxis systems, flagellar assembly pathways, and extracellular polysaccharide biosynthesis, providing a predicted genetic basis for directional migration, motility, surface attachment, and stable rhizosphere colonization.

**FIGURE 8:**
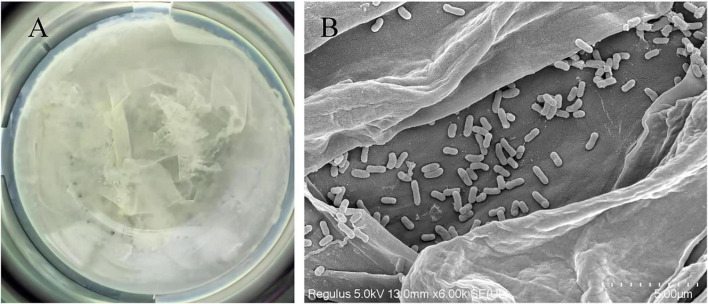
Biofilm formation ability of strain YNK-FB0053 and its colonization on the root surface of Chinese cabbage. **(A)** Biofilm formation; **(B)** colonization of strain YNK-FB0053 on the root surface of Chinese cabbage observed under electron microscopy.

Biofilm formation assays demonstrated that the strain possesses strong biofilm-producing capability. Under stereomicroscopy (Figure 8A), a thick, wrinkled, milky-white biofilm was observed after 24 h of static incubation, with a quantified biomass of 131.4 mg. This phenotypic result supports the genome-based prediction that the strain has genetic potential for surface attachment and biofilm formation.

Pot colonization experiments with Chinese cabbage further confirmed that YNK-FB0053 can successfully colonize root surfaces. Scanning electron microscopy clearly showed bacterial attachment on the root surface (Figure 8B), and plate counting results indicated a rhizosphere colonization density as high as 3.8 × 10^9^ CFU/g. These phenotypic results are highly consistent with the genomic functional annotations, demonstrating that strain YNK-FB0053 possesses excellent rhizosphere colonization ability and can stably persist in the rhizosphere to continuously exert plant growth–promoting effects.

#### 3.11.2 Effects on plant agronomic traits

**FIGURE 9:**
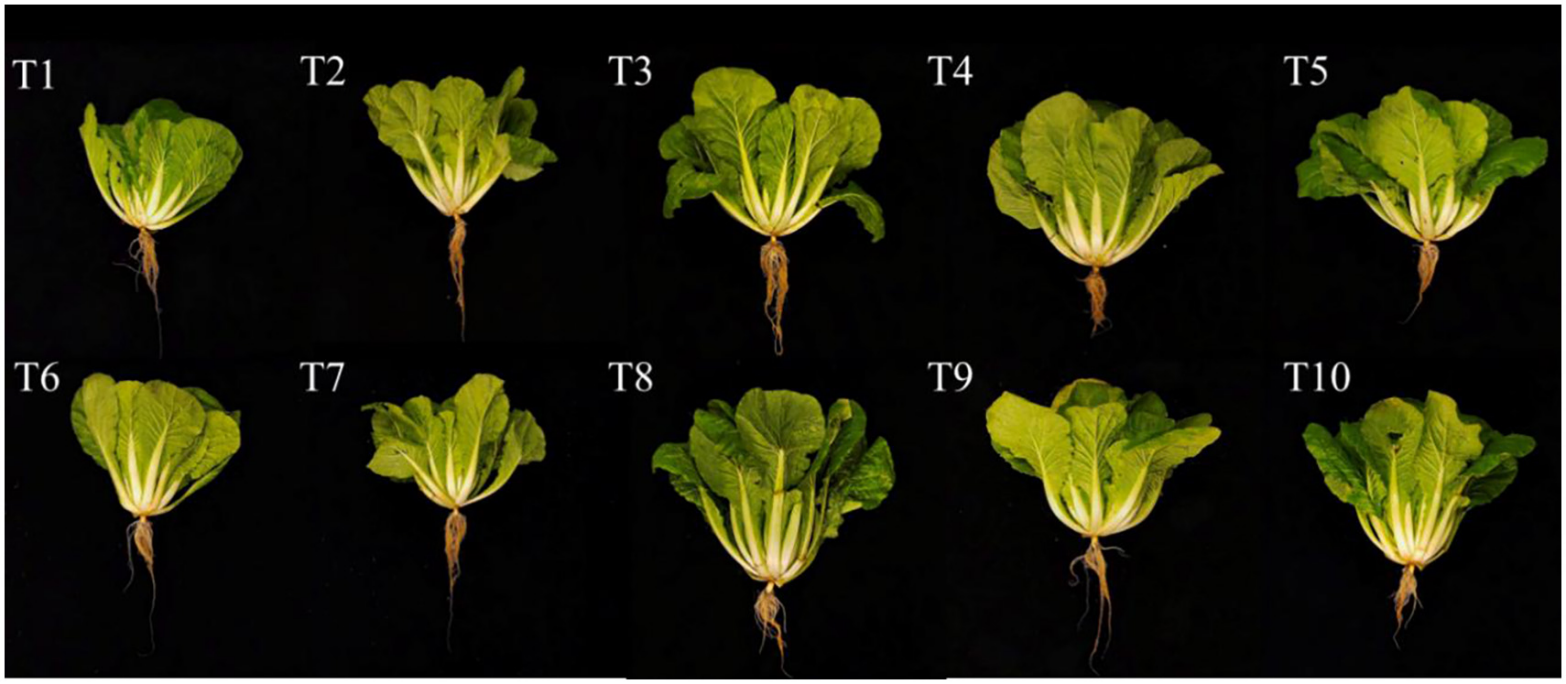
Growth-promoting effect of strain YNK-FB0053 on plants.

**FIGURE 10:**
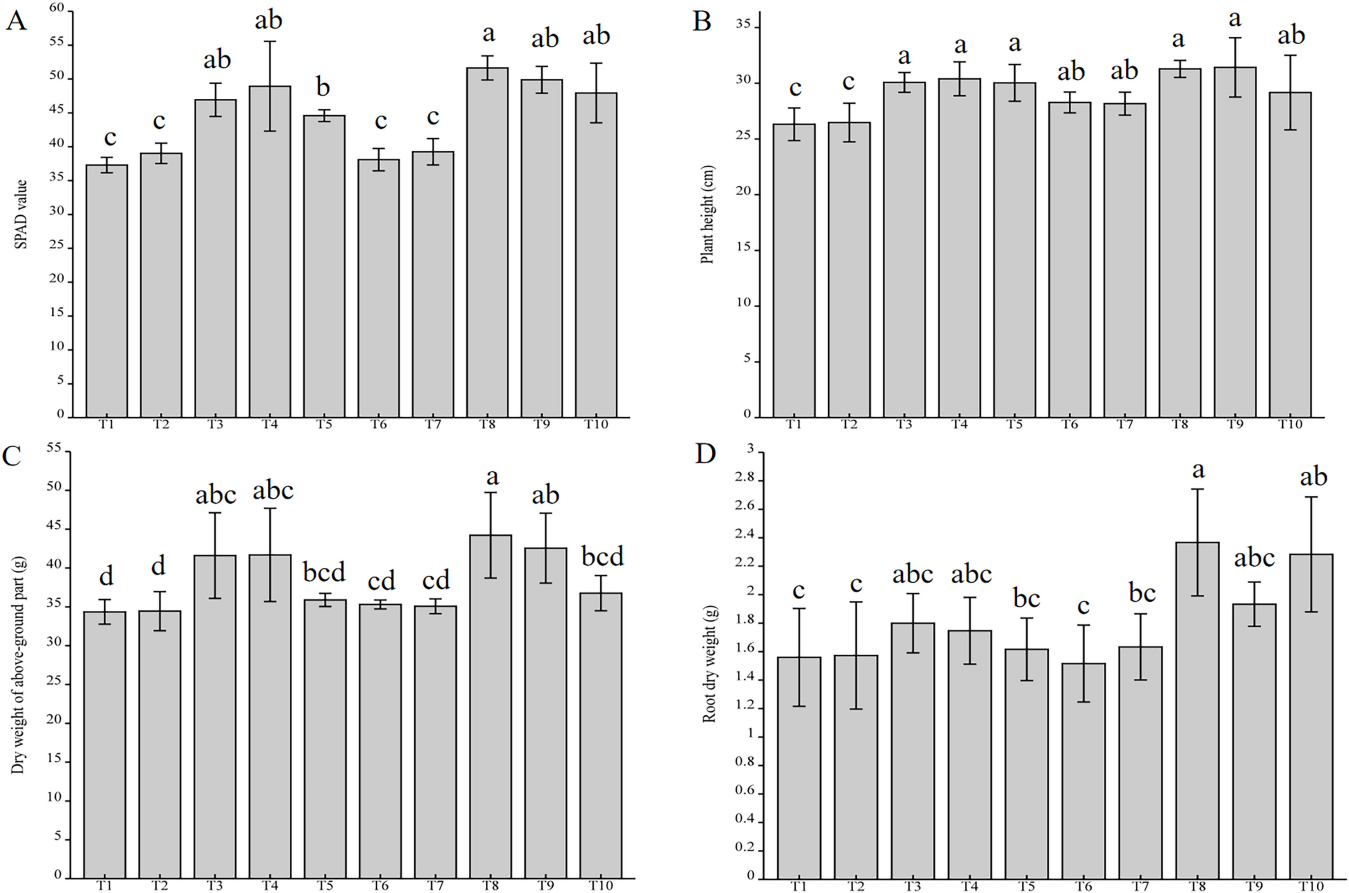
Effects of different treatments on plant growth indices. **(A)** Chlorophyll content (SPAD value); **(B)** plant height; **(C)** shoot dry weight; **(D)**: root dry weight. Bars represent the mean ± standard deviation (SD) of three biological replicates. Different lowercase letters above the bars indicate significant differences among treatments at *P* < 0.05 according to Duncan’s multiple range test.

Agronomic traits including chlorophyll SPAD value, plant height, root length, stem girth, biomass, and leaf number were determined after 50 days of transplantation. The results showed no significant differences in all indices between the water control and medium control, whereas fermentation broth treatments significantly increased chlorophyll SPAD value, plant height, aboveground dry weight, and root dry weight of plants (Figures 9, 10).

Under no sulfur fertilizer application, the fermentation broth increased these indices by 31.39, 15.50, 21.37, and 15.38% compared with control T1, respectively; under sulfur fertilizer application, the increases were 35.54, 11.13, 25.27, and 56.05% compared with control T6, respectively. Overall, the combined application of undiluted fermentation broth and sulfur fertilizer (T8) exhibited the best effect, which was significantly superior to other treatments, indicating that sulfur supply could synergistically enhance the growth-promoting effect with strain function.

#### 3.11.3 Effects on plant nutrient uptake

**FIGURE 11:**
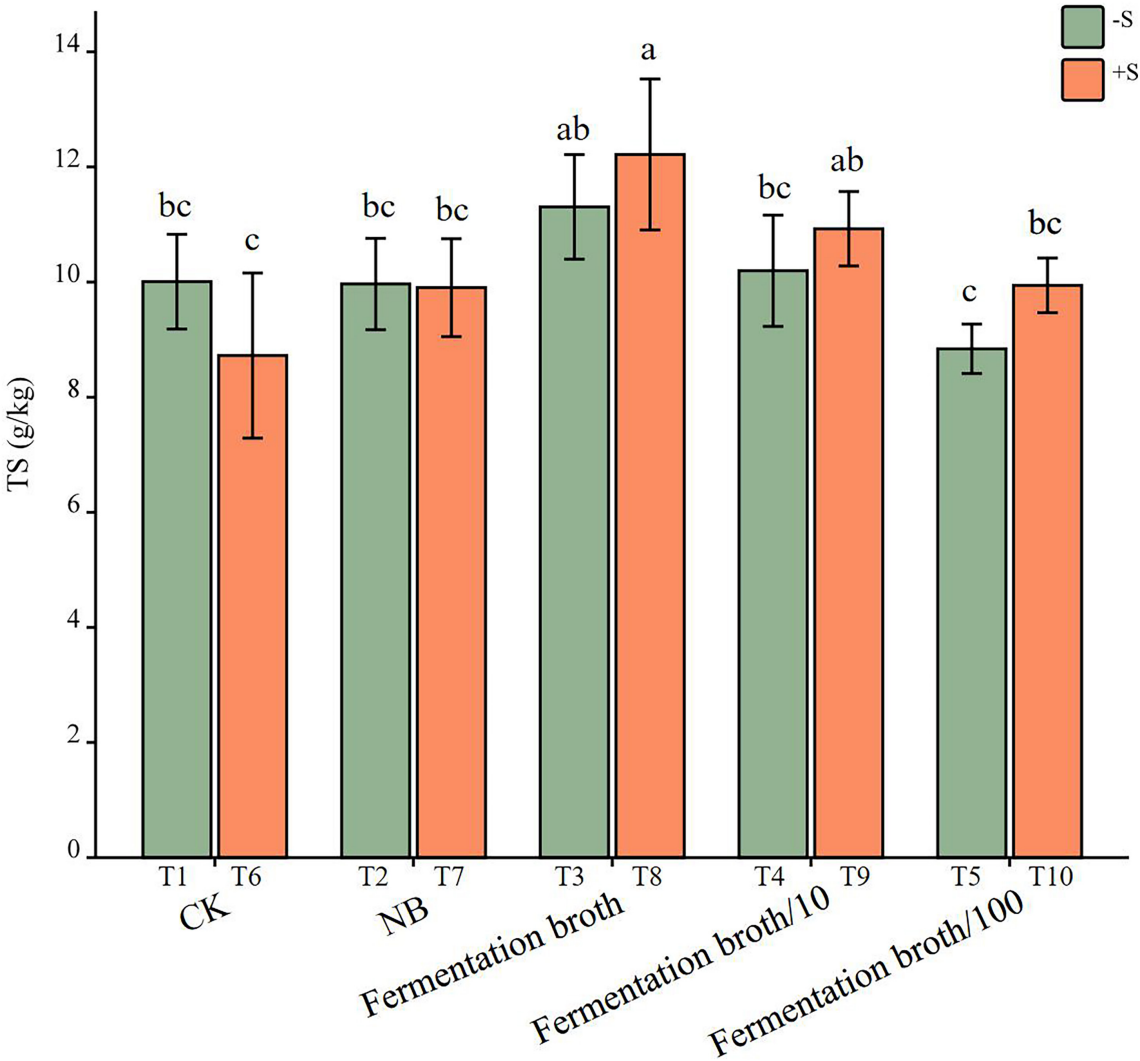
Effects of different treatments on plant sulfur content. Effects of fermentation broth of the strain at different concentrations on plant sulfur content under sulfur-applied (+S) and non-sulfur-applied (–S) conditions. The *x*-axis represents the control and treatments with different concentrations, the *y*-axis represents plant sulfur content, and different colors indicate sulfur-applied (+S) and non-sulfur-applied (–S) groups. Different lowercase letters above the bars indicate significant differences among treatments at *P* < 0.05 according to Duncan’s multiple range test.

Plant nutrient analysis showed that fermentation broth of YNK-FB0053 significantly promoted the accumulation of N, P, K, Fe, and S in plants, especially with the most prominent increase in sulfur. Under no sulfur application, the fermentation broth increased total sulfur content in plants by 12.99% compared with T1; under sulfur application, the increase reached 40.14% compared with T6, with undiluted fermentation broth performing the best (Figure 11). Even in sulfur-limited environments, the strain may improve soil sulfur availability through its predicted sulfur-related metabolic potential and thereby promote plant uptake. Meanwhile, plant nitrogen and phosphorus contents increased synchronously with sulfur uptake, showing an obvious synergistic nutrient absorption effect, which was consistent with the multi-pathway nutrient transformation functions annotated in the genome.

#### 3.11.4 Effects on soil physicochemical properties

Strain fermentation broth significantly improved soil nutrient status. Compared with the control, soil available sulfur and total sulfur contents increased by 68.44 and 71.05% under no sulfur application, and by 80.75 and 68.29% under sulfur application, respectively (Table 2). Meanwhile, soil total phosphorus, hydrolyzable nitrogen, and available potassium contents were also significantly increased. Acidic metabolites potentially generated during sulfur transformation by the strain may regulate soil pH and promote the release of insoluble nutrients, which is consistent with the annotation results of genes predicted to be related to sulfur oxidation and phosphorus solubilization.

**TABLE 2:** Effects of different treatments on soil physical and chemical properties.

Treatment	pH	TN (g/kg)	TP (g/kg)	TK (g/kg)	AN (mg/kg)	AK (mg/kg)	AP (mg/kg)	SOC(%)	AS (mg/kg)	TS (g/kg)
T1	7.30 ± 0.00b	1.76 ± 0.05ab	3.54 ± 0.57e	5.71 ± 0.29b	130.33 ± 5.77bcde	61.33 ± 4.04d	157.73 ± 1.81bc	1.93 ± 0.08b	49.97 ± 2.65d	0.38 ± 0.03b
T2	7.00 ± 0.00e	1.76 ± 0.09ab	3.59 ± 0.56de	5.80 ± 0.10b	128.67 ± 3.21cde	65.33 ± 5.86cd	154.20 ± 0.46c	2.07 ± 0.07ab	44.67 ± 2.54d	0.39 ± 0.02b
T3	6.93 ± 0.06f	1.88 ± 0.05a	4.26 ± 0.08abc	5.94 ± 0.21ab	147.33 ± 1.15a	83.00 ± 1.00a	172.10 ± 0.35a	2.16 ± 0.09ab	77.03 ± 3.57b	0.65 ± 0.15a
T4	7.17 ± 0.06c	1.85 ± 0.06a	4.46 ± 0.29a	5.52 ± 0.14b	137.33 ± 2.52bc	78.33 ± 2.08a	170.10 ± 2.31a	2.14 ± 0.09ab	84.17 ± 2.76a	0.63 ± 0.10a
T5	7.30 ± 0.00b	1.77 ± 0.08ab	4.19 ± 0.29abcd	5.86 ± 0.09ab	139.00 ± 6.93ab	81.33 ± 2.52a	160.90 ± 1.40b	2.02 ± 0.06ab	70.17 ± 7.92b	0.48 ± 0.06b
T6	7.17 ± 0.06c	1.68 ± 0.05b	3.72 ± 0.29cde	5.66 ± 0.16b	123.33 ± 4.93e	63.67 ± 1.15cd	159.00 ± 1.21b	2.03 ± 0.05ab	47.73 ± 1.46d	0.41 ± 0.04b
T7	7.10 ± 0.00d	1.66 ± 0.08b	3.79 ± 0.44bcde	5.59 ± 0.46b	126.00 ± 3.61de	66.00 ± 1.00cd	157.67 ± 1.42bc	2.03 ± 0.13ab	45.40 ± 0.87d	0.47 ± 0.03b
T8	7.30 ± 0.00b	1.79 ± 0.12ab	4.37 ± 0.05ab	6.29 ± 0.33a	134.67 ± 6.81bcd	67.33 ± 1.53bc	161.33 ± 2.16b	2.39 ± 0.47a	86.27 ± 4.01a	0.68 ± 0.10a
T9	7.37 ± 0.06ab	1.73 ± 0.02ab	3.94 ± 0.08abcde	5.89 ± 0.04ab	128.33 ± 7.77cde	68.67 ± 2.52bc	160.80 ± 5.40b	2.30 ± 0.50ab	60.20 ± 4.75c	0.69 ± 0.09a
T10	7.40 ± 0.00a	1.86 ± 0.12a	4.51 ± 0.19a	5.96 ± 0.20ab	128.00 ± 2.65cde	72.00 ± 3.46b	159.10 ± 0.92b	2.12 ± 0.06ab	59.70 ± 5.71c	0.47 ± 0.01b

Values are presented as mean ± SD. Different lowercase letters within the same column indicate significant differences among treatments at *P* < 0.05 according to Duncan’s multiple range test.

#### 3.11.5 Synergistic effects of sulfur fertilizer and strain fermentation broth

**FIGURE 12:**
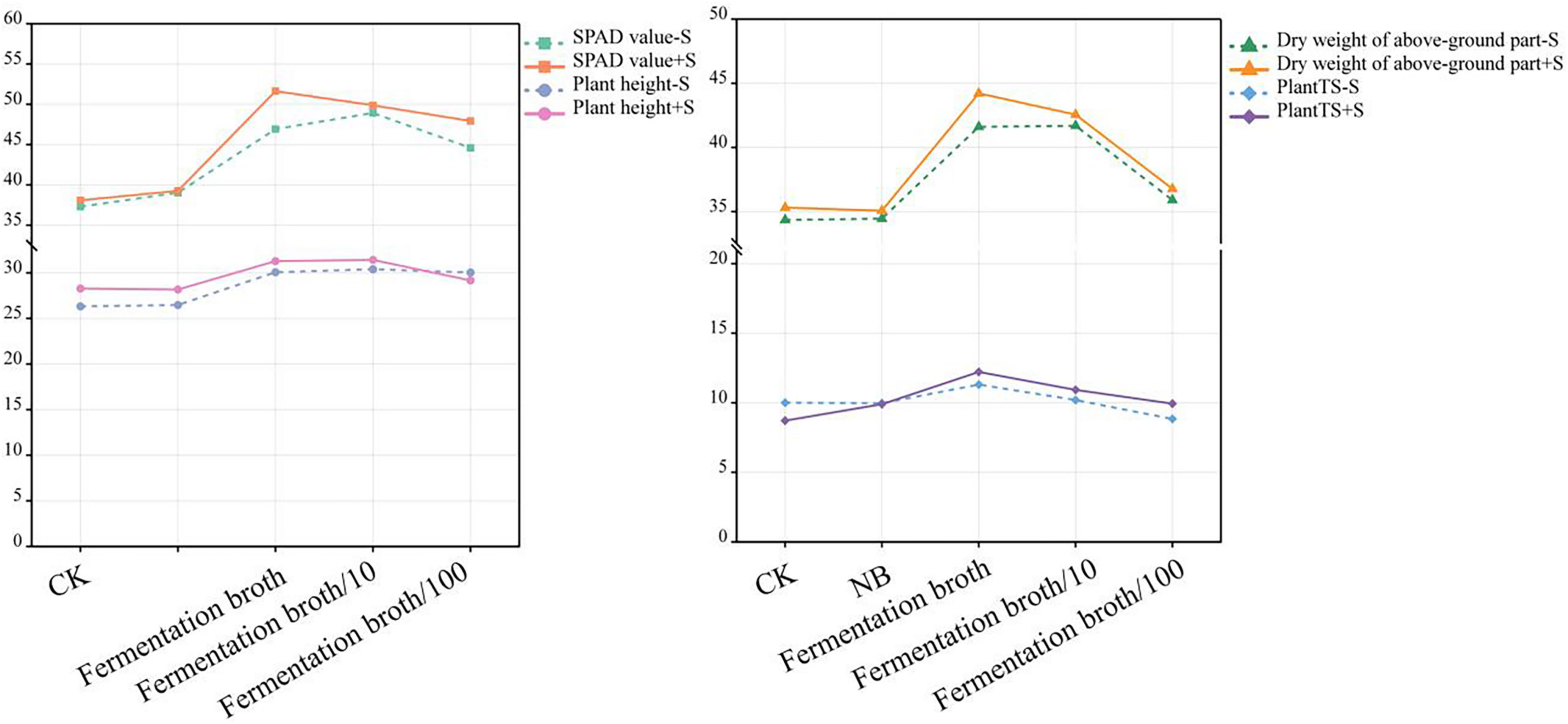
Effects of different treatments on plant chlorophyll content, plant height, shoot dry weight, and plant sulfur content.

The growth-promoting effects of the strain differed significantly between sulfur-fertilized and non-sulfur-fertilized conditions. Under sulfur application, plant chlorophyll content, biomass, and sulfur concentration first increased and then stabilized with rising fermentation broth concentration, with undiluted and low-dilution treatments showing the best effects. Under no sulfur application, the promotion magnitude was lower but still exhibited a positive growth-promoting effect (Figure 12). These results indicated that YNK-FB0053 acts as a critical bridge between soil sulfur cycling and plant sulfur nutrition supply, and the combined application of sulfur fertilizer and the strain achieves optimal growth-promoting performance.

#### 3.11.6 Correlation and principal component analysis

**FIGURE 13:**
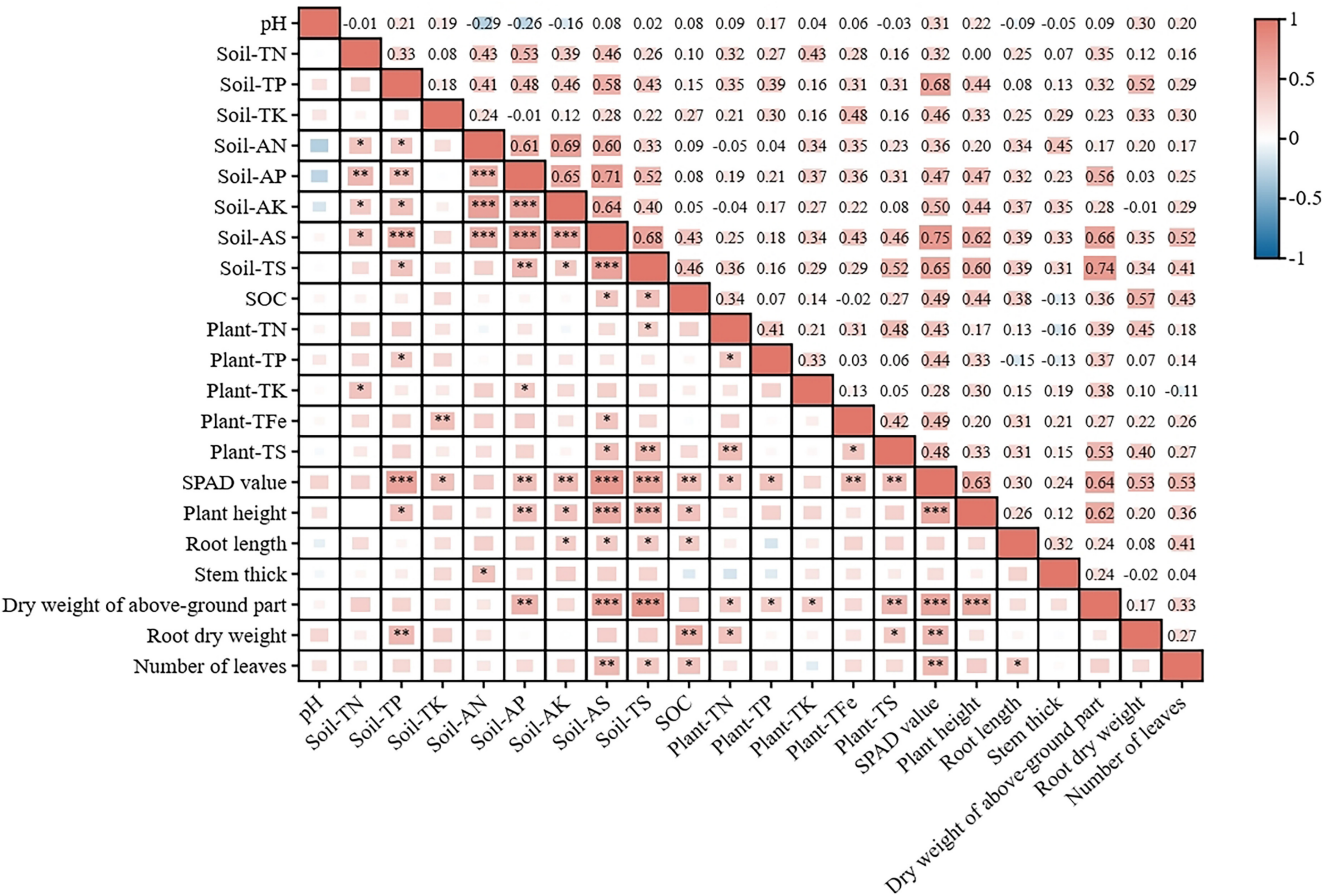
Correlation heatmap between plant agronomic traits, element contents, and soil element contents. Colors ranging from blue to red represent negative and positive correlations, respectively. The magnitude of the correlation coefficient is indicated by the color intensity. Asterisks *, **, and *** indicate significant correlations at the 0.05, 0.01, and 0.001 levels, respectively.

Pearson correlation analysis showed that plant growth indicators were significantly or extremely significantly positively correlated with soil available sulfur, nitrogen, phosphorus, and potassium. Significant positive correlations were also observed between soil available sulfur and total sulfur, as well as between plant sulfur content and nitrogen/phosphorus contents, suggesting that the strain improves overall soil nutrient availability, promotes synergistic nutrient uptake by plants, enhances photosynthesis, and increases biomass (Figure 13).

**FIGURE 14:**
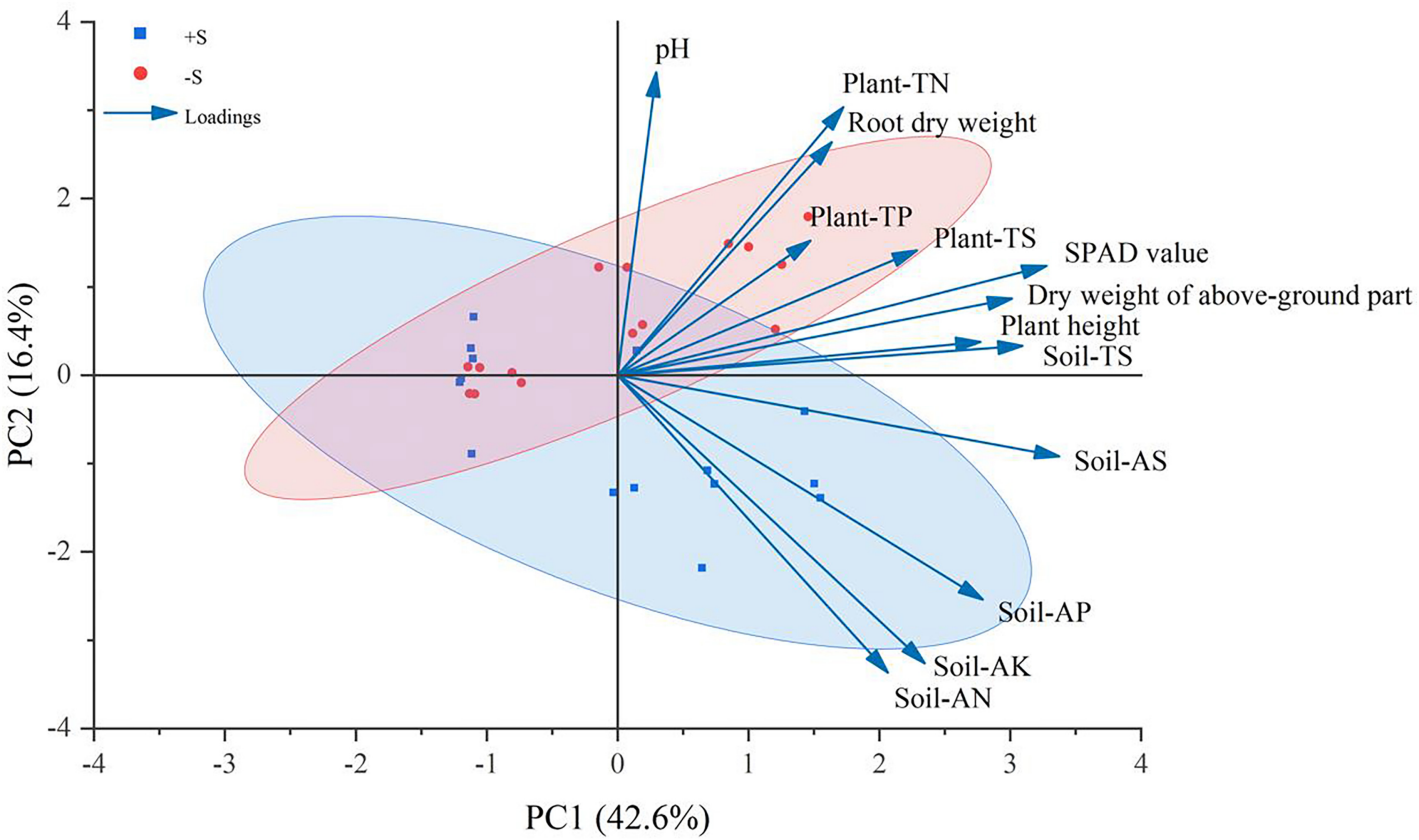
Principal component analysis (PCA) of plant agronomic traits, element contents, and soil element contents.

Principal component analysis (PCA) showed that the cumulative contribution rate of the first two principal components reached 59.0%. PC1 mainly reflected the combined effects of soil nutrient supply and plant growth response, and was highly positively correlated with soil available sulfur, nitrogen, phosphorus, potassium, plant height, SPAD value, and aboveground dry weight. PC2 mainly represented variations in soil pH and plant nitrogen and sulfur accumulation. Treatments with and without sulfur application were clearly separated along PC1, further confirming that sulfur supply and strain function jointly determine plant growth phenotypes, which is highly consistent with genome functional annotation results (Figure 14).

## 4 Discussion

As a key component of rhizosphere microbial communities, the genus *Burkholderia* generally possesses relatively large genomes and high GC contents, features that are often associated with broad metabolic versatility, strong environmental adaptability, and ecological plasticity in complex rhizosphere environments (Depoorter et al., 2016; Eberl and Vandamme, 2016). Consistent with these genus-level characteristics, *Burkholderia gladioli* YNK-FB0053 has a genome size of 8.15 Mb and a GC content of 68.07%. The high quality of the genome assembly was supported by multiple assessment metrics, including 100% genome completeness estimated by both CheckM and BUSCO, together with a low CheckM-estimated contamination rate of 1.68%. Genome annotation identified multiple genomic islands, prophage regions, and numerous genes encoding transmembrane transporters and secreted proteins, suggesting that YNK-FB0053 possesses a flexible genetic repertoire for environmental response, nutrient exchange, and rhizosphere adaptation. In addition, members of the genus *Burkholderia* are frequently enriched in biosynthetic gene clusters associated with secondary metabolite production (Bach et al., 2022). In this study, 19 secondary metabolite biosynthetic gene clusters were identified in YNK-FB0053, most of which showed relatively low similarity to known clusters, and five clusters showed no detectable homologous sequences. Together with previous studies on plant-associated *Burkholderia* strains (Goswami et al., 2016; Mustafa et al., 2019), these findings suggest that YNK-FB0053 may possess diverse secondary metabolic potential, which may contribute to its ecological functional diversity in the rhizosphere. Moreover, the prediction of homoserine lactone-related clusters indicates the presence of a potential quorum-sensing system, which may provide a molecular basis for population-level regulation, biofilm formation, and rhizosphere colonization (Suárez-Moreno et al., 2012).

Regulation of the sulfur cycle represents a central functional feature of strain YNK-FB0053. Genome analysis identified *sqr*, Sox-related genes (*soxA*, *soxB*, *soxC*, *soxD*, and *soxG*), the *cysPUWA* sulfate/thiosulfate transport system, assimilatory sulfate reduction genes (*cysN*, *cysD*, *cysC*, *cysH*, and *cysI*), and organic sulfur utilization genes such as *ssu*, *tau*, and *sfn*. Previous phenotype-based assays showed that sulfate production by YNK-FB0053 reached 38.53–66.54 mg/L after 7 days of cultivation in MST medium (Chen et al., 2026). The presence of *sqr* suggests the potential for sulfide oxidation, whereas Sox-related genes are commonly associated with the oxidation of thiosulfate and other reduced sulfur compounds in sulfur-oxidizing microorganisms (Gregersen et al., 2011; Dahl et al., 2013). However, a complete canonical *soxXYZABCD* cluster and *tsdA* were not annotated in YNK-FB0053; therefore, its sulfur-oxidizing potential is more appropriately interpreted as being associated with an *sqr*-linked sulfide oxidation route and Sox-related sulfur oxidation components, rather than a complete canonical Sox pathway or a *tsdA*-dependent thiosulfate dehydrogenase pathway. This sulfur-related genetic pattern may distinguish YNK-FB0053 from sulfur-oxidizing bacteria that rely primarily on complete Sox systems or *tsdA*-dependent thiosulfate oxidation pathways (Ghosh and Dam, 2009; Frigaard and Dahl, 2008). In addition, the *cysPUWA* sulfate/thiosulfate transport system and assimilatory sulfate reduction genes suggest that YNK-FB0053 may link extracellular sulfur transformation with intracellular sulfate assimilation, forming a potential sulfur oxidation–assimilation route (Kredich, 2008). The detection of *ssu*, *tau*, and *sfn* genes further indicates the potential to utilize diverse organic sulfur sources, such as sulfonates, taurine, and methylated sulfur compounds, which may broaden its ecological role in soil sulfur cycling (Gahan and Schmalenberger, 2014). Moreover, the assimilatory sulfate reduction pathway may contribute to the formation of reduced sulfur metabolites, including sulfide or hydrogen sulfide (H*2*S), during bacterial sulfur metabolism; however, the actual production level and physiological role of these metabolites in the rhizosphere require targeted verification (Kopriva and Rennenberg, 2004). Although H*2*S has been reported as an important signaling molecule involved in seed germination and plant stress responses, its contribution to the YNK-FB0053–plant interaction remains to be experimentally determined (Yusuf et al., 2023; Pourebrahimi et al., 2023; Wang et al., 2023; Wang et al., 2022). Together, these sulfur metabolism-related features provide a focused genomic explanation for the observed increases in plant sulfur accumulation and soil available sulfur content. Through sulfur transformation, YNK-FB0053 may influence the rhizosphere microenvironment and indirectly affect the availability of other nutrients such as phosphorus, supporting a sulfur-driven multi-nutrient interaction in the rhizosphere (Dahl and Friedrich, 2008; Narayan et al., 2023). Nevertheless, detailed sulfur substrates, intermediate metabolites, enzyme activities, and metabolic fluxes should be further verified through targeted biochemical assays.

Beyond sulfur cycling, YNK-FB0053 also exhibits substantial genetic potential for phosphorus, nitrogen, and micronutrient transformation. For phosphorus transformation, the genome harbors a PQQ-dependent glucose dehydrogenase system (*gcd*, *gdh*, and *pqqBCDE*), which may promote the solubilization of insoluble inorganic phosphate through organic acid secretion (Sharma et al., 2013). Consistently, previous experimental results showed that this strain exhibited strong phosphate-solubilizing ability, with soluble phosphorus reaching 567.47 mg/L (Chen et al., 2026). In addition, genes such as *glpQ*, *ppa*, and *ppx–gppA* may confer the potential for organic phosphorus mineralization and polyphosphate utilization (Richardson et al., 2011), whereas the PstSCAB transport system and Pho regulon may enhance its adaptation to phosphorus-limited conditions (Santos–Beneit, 2015; Alori et al., 2017).

In nitrogen metabolism, the canonical nitrogenase structural genes *nifH*, *nifD*, and *nifK* were not detected in this study; therefore, YNK-FB0053 should not be described as having confirmed nitrogen-fixing ability. The *narK* gene encodes nitrate/nitrite transmembrane transporters (Kraft et al., 2011), *nirB* encodes an NADH-dependent nitrite reductase (Simon and Klotz, 2013), and *glnA*, *gdhA*, and *glnB* are involved in the GS–GOGAT/glutamate dehydrogenase nitrogen assimilation and regulatory system (Merrick and Edwards, 1995). Together, these genes suggest that YNK-FB0053 has the potential for nitrate transport, nitrite reduction, and ammonium assimilation, which may contribute to improved nitrogen utilization efficiency in the rhizosphere (Kraft et al., 2011). In addition, the cyanate conversion pathway mediated by *cynS* and *cynT* may allow this strain to use cyanate as an alternative nitrogen source under nitrogen-limited conditions (Flores and Herrero, 2005). The *iscU* gene is involved in Fe–S cluster biosynthesis (Py and Barras, 2010), which may help maintain the structural and functional stability of some nitrogen metabolism-related enzymes, but it should not be regarded as direct evidence for nitrogen fixation.

Regarding micronutrient regulation, YNK-FB0053 harbors multiple genes associated with zinc homeostasis and iron acquisition. The *zur* gene may participate in the regulation of zinc uptake and efflux (Mikhaylina et al., 2018), whereas *zntA* and *zntB* are associated with intracellular zinc homeostasis (Barisch et al., 2018). In addition, the TonB–ExbB–ExbD system provides energy for outer-membrane transport processes involving zinc and other metal ions (Schauer et al., 2008), collectively supporting the predicted capacity of YNK-FB0053 for zinc regulation and its potential contribution to plant zinc nutrition (Blindauer, 2015). For iron acquisition, YNK-FB0053 carries candidate genes related to siderophore export and high-affinity iron transport. Specifically, *entS* may participate in siderophore export (Miethke and Marahiel, 2007), *afuABC* may mediate high-affinity Fe^3+^ uptake (Andrews et al., 2003), and *furB* may contribute to iron homeostasis regulation (Fillat, 2014). In addition, high-affinity iron transport-related genes such as *efeU* and *ftrA*, together with AraC-type transcriptional regulators, may enhance iron acquisition under iron-limited conditions. Previous CAS assay results showed that YNK-FB0053 exhibited siderophore-producing activity, with a siderophore activity of 60.76% (Chen et al., 2026), providing phenotypic support for the genomic prediction. Siderophore production may improve microbial competitiveness in the rhizosphere, contribute to pathogen suppression, and facilitate plant growth by increasing iron availability (Wang et al., 2013). However, the specific siderophore types and production levels of YNK-FB0053 require further chemical identification and quantitative analysis.

In addition to nutrient transformation, YNK-FB0053 may contribute to plant growth promotion and the mitigation of continuous cropping stress through phytohormone-related pathways. Genome annotation revealed genes associated with tryptophan biosynthesis and multiple tryptophan-dependent indole-3-acetic acid (IAA) biosynthetic routes. Tryptophan serves as a major precursor for microbial IAA biosynthesis, whereas ALDH and *amiE* may participate in different IAA conversion steps (Yang et al., 2016; Duca et al., 2014; Hou et al., 2017). The coexistence of these tryptophan-dependent pathways may support IAA production under variable rhizosphere conditions, thereby contributing to root development, nutrient acquisition, and plant growth promotion (Backer et al., 2018; Lugtenberg and Kamilova, 2009).

In terms of phenolic compound degradation, YNK-FB0053 carries genes associated with aromatic compound metabolism. The *ben* genes are related to benzoate metabolism (Harwood and Parales, 1996; Clark et al., 2004), *cat* genes may mediate ortho- and meta-cleavage of catechol (Kukor et al., 1988), and the *pca* cluster may support degradation via the β-ketoadipate pathway (Kamimura et al., 2017). The *paa* cluster further suggests the potential to degrade phenylacetic acid-type aromatic compounds (Ismail et al., 2003). These predicted pathways may contribute to phenolic compound turnover in the rhizosphere and help alleviate continuous cropping stress, but direct degradation assays are needed for confirmation.

Rhizosphere colonization is essential for sustained functional performance. YNK-FB0053 contains complete chemotaxis (*che*) and flagellar assembly (*flh*, *fli*, *flg*, *mot*) systems. Root exudates can be sensed by MCP receptors (Scharf et al., 2016), driving directional migration. Flagella provide structural support for motility and biofilm formation (Compant et al., 2019), while CheY-mediated signaling regulates motility behavior (Bi and Sourjik, 2018). Extracellular polysaccharides enhance adhesion and biofilm stability (Beauregard et al., 2013; Flemming and Wingender, 2010; Bashan and de-Bashan, 2010), collectively enabling strong colonization ability (Gupta Sood, 2003).

Furthermore, YNK-FB0053 possesses multiple heavy metal resistance systems (Cus, Czc, Chr, Ars), including CusRS–CusCFBA (Bondarczuk and Piotrowska-Seget, 2013), CzcABC (Rajkumar et al., 2012), ChrA/ChrR (Cervantes et al., 2001), and arsRBC modules, ensuring metabolic stability under heavy metal stress and supporting its application in contaminated soils (Wang et al., 2025; Okpara-Elom et al., 2024).

Pot experiments provided plant-level evidence for the growth-promoting effect of YNK-FB0053 and its interaction with sulfur supply. Previous phenotype-based assays reported sulfate production, phosphate solubilization, and CAS-positive siderophore activity in this strain (Chen et al., 2026). In this study, YNK-FB0053 fermentation broth significantly increased chlorophyll content, plant height, and biomass of Chinese cabbage under both sulfur-fertilized and non-sulfur-fertilized conditions, with stronger effects observed under sulfur application. This suggests that YNK-FB0053 may promote plant growth by enhancing soil sulfur transformation and sulfur fertilizer use efficiency, rather than relying solely on external sulfur input, consistent with the ecological role of sulfur-oxidizing bacteria in linking soil sulfur pools with plant sulfur nutrition (Kertesz and Mirleau, 2004). Plant nutrient analysis further showed increased sulfur accumulation, which was positively associated with nitrogen and phosphorus uptake. Adequate sulfur supply can enhance nitrogen assimilation and photosynthetic metabolism (Hawkesford and De Kok, 2006), thereby promoting coordinated nutrient uptake and biomass accumulation. Even without sulfur fertilization, YNK-FB0053 still promoted plant growth, suggesting its potential to mobilize native soil sulfur pools under sulfur-limited conditions (Liu et al., 2024). The consistency of responses across plant growth traits, plant nutrient accumulation, and rhizosphere nutrient changes indicates that the three-replicate controlled pot experiment provided sufficient support for treatment-level evaluation of YNK-FB0053 under greenhouse conditions. Broader greenhouse or field evaluations would further help assess the agronomic stability and practical applicability of this strain under variable agricultural environments.

Soil physicochemical analysis further confirmed that YNK-FB0053 significantly increased available sulfur and other nutrients, showing synergistic effects with sulfur fertilization (Mao et al., 2022; Feng et al., 2026). Correlation analysis indicated that available sulfur plays a central regulatory role in soil nutrient systems, supporting its importance as the “fourth major nutrient” (Li et al., 2022).

In addition, members of the genus *Burkholderia* display considerable ecological and functional diversity; however, some species or strains have been associated with opportunistic infections in humans, animals, or plants (Depoorter et al., 2016; Eberl and Vandamme, 2016). Therefore, although YNK-FB0053 showed strong rhizosphere colonization ability and plant growth-promoting potential in this study, its use as a candidate agricultural microbial inoculant requires systematic biosafety evaluation. Future studies should include antibiotic susceptibility testing, assessment of virulence-associated genes, safety evaluation for non-target organisms, and analysis of environmental persistence and dissemination risks. For species such as *Burkholderia gladioli*, which may carry potential opportunistic pathogenicity risks, genome-based safety assessment should be combined with application-scenario-based risk evaluation to ensure the feasibility and safety of agricultural use.

## 5 Conclusion

In this study, a high-quality complete genome of *Burkholderia gladioli* YNK-FB0053 was obtained using combined Illumina NovaSeq and PacBio Sequel sequencing. The assembly consisted of two circular chromosomes, with a genome size of 8,148,635 bp and a GC content of 68.07%, and both CheckM and BUSCO analyses indicated 100% completeness. Functional annotation predicted candidate genes related to sulfur oxidation, phosphorus transformation, inorganic nitrogen transformation and assimilation, siderophore-related iron acquisition, IAA biosynthesis, chemotaxis, biofilm formation, and environmental adaptation. In particular, *sqr* and Sox-related genes (*soxA*, *soxB*, *soxC*, *soxD*, and *soxG*), together with sulfate/thiosulfate transport and sulfur assimilation genes, provide a sulfur-centered genomic basis for the predicted sulfur-oxidizing potential of this strain. Phenotypic and pot experiments further showed that the strain has strong biofilm formation and rhizosphere colonization capacities and can promote Chinese cabbage growth, nutrient uptake, and rhizosphere nutrient availability, particularly available sulfur. Overall, YNK-FB0053 is a plant growth-promoting candidate strain with predicted sulfur oxidation and multi-nutrient mobilization potential and may provide a strain resource and theoretical basis for microbe-mediated soil sulfur cycling research and new biofertilizer development. Further biochemical verification of sulfur metabolic activity and systematic biosafety evaluation are required before field-scale agricultural application.

## Data Availability

The datasets presented in this study can be found in online repositories. The names of the repository/repositories and accession number(s) can be found in the article/Supplementary material.
