# Supplementary material for: Genome-wide analysis and growth-promoting potential of Burkholderia gladioli YNK-FB0053
Source: Front Microbiol. 2026 Jul 9;17:1858481. doi: 10.3389/fmicb.2026.1858481 (PMC13391558; doi:10.3389/fmicb.2026.1858481)
Supplement: Supplementary file 1 [file Table_1.pdf]

Genome-Wide Analysis and Growth-Promoting Potential of *Burkholderia gladioli* YNK-FB0053

Dexian Wu[1,2,#], Yan Chen[2,#] , Te Pu[2], Zhufeng Shi[2], Weihua Pei[2], Jiacai Tang[3], Jinbi Hu[1,2]

Qibin Chen[1,*] Peiwen Yang[2,*]

[1] College of Plant Protection, Yunnan Agricultural University, Kunming, China

[2]Institute of Agricultural Environment and Resources, Yunnan Academy of Agricultural Sciences, Kunming, China

[3] Agricultural and Rural Development Service Center, Dalongtan Township, Eshan Yi Autonomous County, Yunnan, China
*Corresponding Author:

*Qibin Chen:tclass99@126.com *Peiwen Yang:pwyang2000@126.com

#These authors have contributed equally to this work and share first authorship.

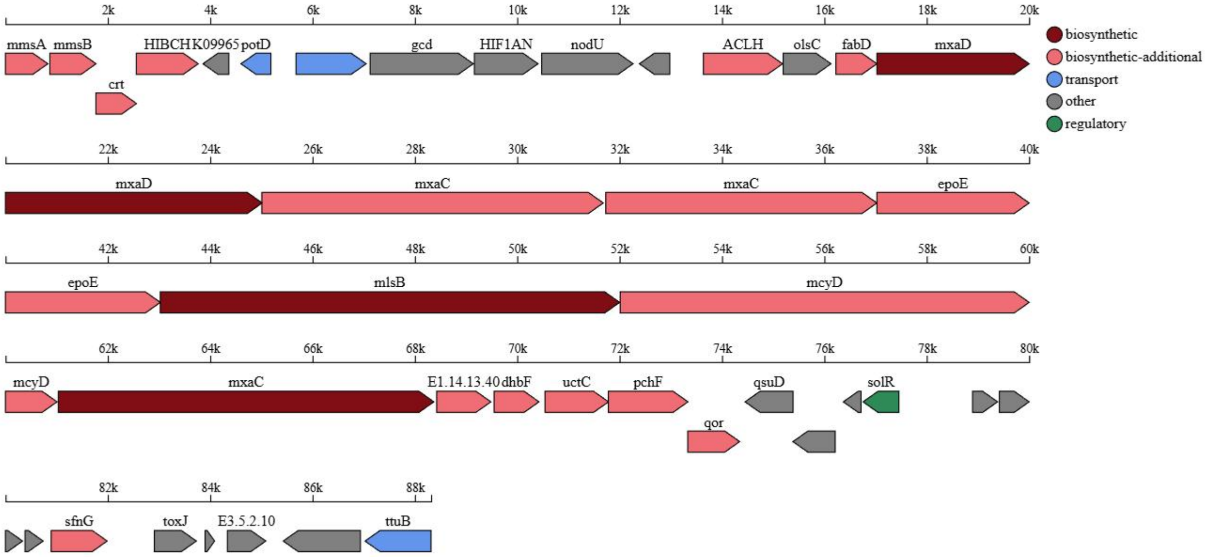

Figure S1. Linear map of secondary metabolite synthesis gene clusters in YNK-FB0053 strain.The map displays all genes within the predicted gene cluster; distinct colors of annotated genes indicate their functional categories in the secondary metabolite biosynthetic gene cluster.

Table S1. Gene clusters of secondary metabolites of *YNK-FB0053* strain

Cluster ID	bp	Gene number	Cluster type	Most similar cluster (%)
cluster1	88371	39	T1PKS	enacyloxin IIa (100)
cluster2	60528	37	NRPS	rhizomide A / B / C (100)
cluster3	20616	22	hserlactone	-
cluster4	41687	38	phosphonate	phosphinothricintripeptide (6)
cluster5	43883	25	T1PKS	-
cluster6	9719	8	RiPP-like	-
cluster7	55207	33	NRPS	pyoverdin (1)
cluster8	81834	39	NRPS	gramibactin (46)
cluster9	80424	47	NRPS	nunapeptin / nunamycin (71)
cluster10	52991	40	NRPS	sulfazecin (100)
cluster11	15774	18	terpene	-
cluster12	22222	20	redox-cofactor	lankacidin C (13)
cluster13	22492	19	terpene	-
cluster14	55049	33	NRPS	icosalide A / B (100)
cluster15	41834	38	RiPP-like	barbamide (41)
cluster1	47654	32	T1PKS	lipopolysaccharide (5)
cluster2	63229	40	RiPP-like	napsamycin A / B / C / D / mureidomycin A / B (6)
cluster3	20841	20	terpene	lasalocid (5)
cluster4	73505	33	NRPS	nunapeptin / nunamycin (8)

Table S2. Genes associated with sulfur cycling in the whole genome of *Burkholderia gladioli* YNK-FB0053

Gene ID	Gene name	KO ID	KO Description	Location
gene1786	cysA	K02045	sulfate/thiosulfate transport system ATP-binding protein [EC:7.3.2.3]	Chromosome1
gene4148	cysC	K00860	adenylylsulfate kinase [EC:2.7.1.25]	Chromosome2
gene5540	cysC	K00860	adenylylsulfate kinase [EC:2.7.1.25]	Chromosome2
gene3091	cysD	K00957	sulfate adenylyltransferase subunit 2 [EC:2.7.7.4]	Chromosome1
gene5523	cysD	K00957	sulfate adenylyltransferase subunit 2 [EC:2.7.7.4]	Chromosome2
gene2580	cysE	K00640	serine O-acetyltransferase [EC:2.3.1.30]	Chromosome1
gene6226	cysE	K00640	serine O-acetyltransferase [EC:2.3.1.30]	Chromosome2

gene1418	cysH	K00390	phosphoadenosine phosphosulfate reductase [EC:1.8.4.8 1.8.4.10]	Chromosome1
gene3093	cysH	K00390	phosphoadenosine phosphosulfate reductase [EC:1.8.4.8 1.8.4.10]	Chromosome1
gene3092	cysH	K00390	phosphoadenosine phosphosulfate reductase [EC:1.8.4.8 1.8.4.10]	Chromosome1
gene6528	cysH	K00390	phosphoadenosine phosphosulfate reductase [EC:1.8.4.8 1.8.4.10]	Chromosome2
gene1417	cysI	K00381	sulfite reductase (NADPH) hemoprotein beta-component [EC:1.8.1.2]	Chromosome1
gene3094	cysI	K00381	sulfite reductase (NADPH) hemoprotein beta-component [EC:1.8.1.2]	Chromosome1
gene6529	cysI	K00381	sulfite reductase (NADPH) hemoprotein beta-component [EC:1.8.1.2]	Chromosome2
gene5522	cysN	K00956	sulfate adenylyltransferase subunit 1 [EC:2.7.7.4]	Chromosome2
gene3561	cysP	K02048	sulfate/thiosulfate transport system substrate-binding protein	Chromosome1
gene1784	cysU	K02046	sulfate/thiosulfate transport system permease protein	Chromosome1
gene1785	cysW	K02047	sulfate/thiosulfate transport system permease protein	Chromosome1
gene2140	sqr	K17218	sulfide:quinone oxidoreductase [EC:1.8.5.4]	Chromosome1
gene3496	tauA	K15551	taurine transport system substrate-binding protein	Chromosome1
gene3495	tauB	K10831	taurine transport system ATP-binding protein [EC:7.6.2.7]	Chromosome1
gene3494	tauC	K15552	taurine transport system permease protein	Chromosome1
gene1543	ssuA	K15553	sulfonate transport system substrate-binding protein	Chromosome1
gene3290	ssuA	K15553	sulfonate transport system substrate-binding protein	Chromosome1
gene4128	ssuA	K15553	sulfonate transport system substrate-binding protein	Chromosome2
gene4142	ssuA	K15553	sulfonate transport system substrate-binding protein	Chromosome2
gene4143	ssuA	K15553	sulfonate transport system substrate-binding protein	Chromosome2
gene4522	ssuA	K15553	sulfonate transport system substrate-binding protein	Chromosome2
gene4727	ssuA	K15553	sulfonate transport system substrate-binding protein	Chromosome2
gene4959	ssuA	K15553	sulfonate transport system substrate-binding protein	Chromosome2
gene5276	ssuA	K15553	sulfonate transport system substrate-binding protein	Chromosome2
gene5280	ssuA	K15553	sulfonate transport system substrate-binding protein	Chromosome2
gene6005	ssuA	K15553	sulfonate transport system substrate-binding protein	Chromosome2
gene1719	ssuB	K15555	sulfonate transport system ATP-binding protein [EC:7.6.2.14]	Chromosome1

gene1720	ssuB	K15555	sulfonate transport system ATP-binding protein [EC:7.6.2.14]	Chromosome1
gene4523	ssuB	K15555	sulfonate transport system ATP-binding protein [EC:7.6.2.14]	Chromosome2
gene4681	ssuB	K15555	sulfonate transport system ATP-binding protein [EC:7.6.2.14]	Chromosome2
gene4961	ssuB	K15555	sulfonate transport system ATP-binding protein [EC:7.6.2.14]	Chromosome2
gene5278	ssuB	K15555	sulfonate transport system ATP-binding protein [EC:7.6.2.14]	Chromosome2
gene1718	ssuC	K15554	sulfonate transport system permease protein	Chromosome1
gene4524	ssuC	K15554	sulfonate transport system permease protein	Chromosome2
gene4960	ssuC	K15554	sulfonate transport system permease protein	Chromosome2
gene5277	ssuC	K15554	sulfonate transport system permease protein	Chromosome2
gene1717	ssuD	K04091	alkanesulfonate monooxygenase [EC:1.14.14.5 1.14.14.34]	Chromosome1
gene1870	ssuD	K04091	alkanesulfonate monooxygenase [EC:1.14.14.5 1.14.14.34]	Chromosome1
gene5274	ssuD	K04091	alkanesulfonate monooxygenase [EC:1.14.14.5 1.14.14.34]	Chromosome2
gene4627	ssuE	K00299	FMN reductase [EC:1.5.1.38]	Chromosome2
gene6717	ssuE	K00299	FMN reductase [EC:1.5.1.38]	Chromosome2
gene3831	sfnG	K17228	dimethylsulfone monooxygenase [EC:1.14.14.35]	Chromosome2
gene5087	sseA	K01011	thiosulfate/3-mercaptopyruvate sulfurtransferase [EC:2.8.1.1 2.8.1.2]	Chromosome2
gene2275	dmdB	K20034	3-(methylthio)propionyl---CoA ligase [EC:6.2.1.44]	Chromosome1
gene4464	dmdB	K20034	3-(methylthio)propionyl---CoA ligase [EC:6.2.1.44]	Chromosome2
gene0976	dmdC	K20035	3-(methylsulfanyl)propanoyl-CoA dehydrogenase [EC:1.3.99.41]	Chromosome1
gene3352	dmdC	K20035	3-(methylsulfanyl)propanoyl-CoA dehydrogenase [EC:1.3.99.41]	Chromosome1
gene2158	metC	K01760	cysteine-S-conjugate beta-lyase [EC:4.4.1.13]	Chromosome1
gene5270	metC	K01760	cysteine-S-conjugate beta-lyase [EC:4.4.1.13]	Chromosome2
gene2095	metE	K00549	5-methyltetrahydropteroyltriglutamate--homocysteine methyltransferase [EC:2.1.1.14]	Chromosome1
gene4629	metE	K00549	5-methyltetrahydropteroyltriglutamate--homocysteine methyltransferase [EC:2.1.1.14]	Chromosome2
gene0215	metF	K00297	methylenetetrahydrofolate reductase (NADH) [EC:1.5.1.54]	Chromosome1
gene0971	metN	K02071	D-methionine transport system ATP-binding protein	Chromosome1
gene0972	metI	K02072	D-methionine transport system permease protein	Chromosome1
gene0973	metQ	K02073	D-methionine transport system substrate-binding protein	Chromosome1

gene3449	metQ	K02073	D-methionine transport system substrate-binding protein	Chromosome1
gene4517	metQ	K02073	D-methionine transport system substrate-binding protein	Chromosome2
gene5916	metQ	K02073	D-methionine transport system substrate-binding protein	Chromosome2
gene3055	metG	K01874	methionyl-tRNA synthetase [EC:6.1.1.10]	Chromosome1
gene3532	metH	K00548	5-methyltetrahydrofolate--homocysteine methyltransferase [EC:2.1.1.13]	Chromosome1
gene3533	metH	K00548	5-methyltetrahydrofolate--homocysteine methyltransferase [EC:2.1.1.13]	Chromosome1
gene6256	metH	K00548	5-methyltetrahydrofolate--homocysteine methyltransferase [EC:2.1.1.13]	Chromosome2
gene3709	metK	K00789	S-adenosylmethionine synthetase [EC:2.5.1.6]	Chromosome1
gene3726	metX	K00641	homoserine O-acetyltransferase/O-succinyltransferase [EC:2.3.1.31 2.3.1.46]	Chromosome1
gene3727	metX	K00641	homoserine O-acetyltransferase/O-succinyltransferase [EC:2.3.1.31 2.3.1.46]	Chromosome1
gene4584	metX	K00641	homoserine O-acetyltransferase/O-succinyltransferase [EC:2.3.1.31 2.3.1.46]	Chromosome2
gene4319	metY	K01740	O-acetylhomoserine (thiol)-lyase [EC:2.5.1.49]	Chromosome2
gene4626	metR	K03576	LysR family transcriptional regulator, regulator for metE and metH	Chromosome2
gene4825	metZ	K10764	O-succinylhomoserine sulfhydrylase [EC:2.5.1.-]	Chromosome2
gene4642	soxC	K17225	sulfane dehydrogenase subunit SoxC	Chromosome2
gene4643	soxD	K22622	S-disulfanyl-L-cysteine oxidoreductase SoxD [EC:1.8.2.6]	Chromosome2
gene5938	soxR	K13639	MerR family transcriptional regulator, redox-sensitive transcriptional activator SoxR	Chromosome2
gene6762	soxB	K00303	sarcosine oxidase, subunit beta [EC:1.5.3.24 1.5.3.1]	Chromosome2
gene6763	soxD	K00304	sarcosine oxidase, subunit delta [EC:1.5.3.24 1.5.3.1]	Chromosome2
gene6764	soxA	K00302	sarcosine oxidase, subunit alpha [EC:1.5.3.24 1.5.3.1]	Chromosome2
gene6765	soxG	K00305	sarcosine oxidase, subunit gamma [EC:1.5.3.24 1.5.3.1]	Chromosome2

Table S3. Genes associated with phosphate solubilization in the complete genome of *Burkholderia gladioli* YNK-FB0053

Gene ID	Gene name	KO ID	KO Description	Location
gene0981	gcd	K00117	quinoprotein glucose dehydrogenase [EC:1.1.5.2]	Chromosome1
gene3805	gcd	K00117	quinoprotein glucose dehydrogenase [EC:1.1.5.2]	Chromosome2
gene4348	gcd	K00117	quinoprotein glucose dehydrogenase [EC:1.1.5.2]	Chromosome2
gene3626	gdh	K00034	glucose 1-dehydrogenase [EC:1.1.1.47]	Chromosome1
gene4040	gdh	K00034	glucose 1-dehydrogenase [EC:1.1.1.47]	Chromosome2
gene5002	gdh	K00034	glucose 1-dehydrogenase [EC:1.1.1.47]	Chromosome2
gene6012	pqqB	K06136	pyrroloquinoline quinone biosynthesis protein B	Chromosome2
gene6011	pqqC	K06137	pyrroloquinoline-quinone synthase [EC:1.3.3.11]	Chromosome2
gene6010	pqqD	K06138	pyrroloquinoline quinone biosynthesis protein D	Chromosome2
gene6009	pqqE	K06139	PqqA peptide cyclase [EC:1.21.98.4]	Chromosome2
gene3038	ppa	K01507	inorganic pyrophosphatase [EC:3.6.1.1]	Chromosome1
gene1347	ppx-gppA	K01524	exopolyphosphatase / guanosine-5'-triphosphate,3'-diphosphate pyrophosphatase [EC:3.6.1.11 3.6.1.40]	Chromosome1
gene2242	TC.PIT	K03306	inorganic phosphate transporter, PiT family	Chromosome1
gene6667	TC.PIT	K03306	inorganic phosphate transporter, PiT family	Chromosome2
gene1339	pstS	K02040	phosphate transport system substrate-binding protein	Chromosome1
gene1340	pstC	K02037	phosphate transport system permease protein	Chromosome1
gene1341	pstA	K02038	phosphate transport system permease protein	Chromosome1
gene1342	pstB	K02036	phosphate transport system ATP-binding protein [EC:7.3.2.1]	Chromosome1
gene1343	phoU	K02039	phosphate transport system protein	Chromosome1
gene1344	phoB	K07657	two-component system, OmpR family, phosphate regulon response regulator PhoB	Chromosome1
gene5291	phoB	K07657	two-component system, OmpR family, phosphate regulon response regulator PhoB	Chromosome2

gene5456	phoB	K07657	two-component system, OmpR family, phosphate regulon response regulator PhoB	Chromosome2
gene3327	phoH	K06217	phosphate starvation-inducible protein PhoH and related proteins	Chromosome1
gene1345	phoR	K07636	two-component system, OmpR family, phosphate regulon sensor histidine kinase PhoR [EC:2.7.13.3]	Chromosome1
gene1592	phoR	K07636	two-component system, OmpR family, phosphate regulon sensor histidine kinase PhoR [EC:2.7.13.3]	Chromosome1
gene4887	phnF	K02043	GntR family transcriptional regulator, phosphonate transport system regulatory protein	Chromosome2
gene0348	ugpB	K05813	sn-glycerol 3-phosphate transport system substrate-binding protein	Chromosome1
gene0349	ugpA	K05814	sn-glycerol 3-phosphate transport system permease protein	Chromosome1
gene0350	ugpE	K05815	sn-glycerol 3-phosphate transport system permease protein	Chromosome1
gene0351	ugpC	K05816	sn-glycerol 3-phosphate transport system ATP-binding protein [EC:7.6.2.10]	Chromosome1
gene0352	glpQ	K01126	glycerophosphoryl diester phosphodiesterase [EC:3.1.4.46]	Chromosome1
gene4903	glpQ	K01126	glycerophosphoryl diester phosphodiesterase [EC:3.1.4.46]	Chromosome2

Table S4. Genes associated with nitrogen cycling in the complete genome of *Burkholderia gladioli* YNK-FB0053

Gene ID	Gene name	KO ID	KO Description	Location
gene2682	iscU	K04488	nitrogen fixation protein NifU and related proteins	Chromosome1
gene0594	narK	K02575	MFS transporter, NNP family, nitrate/nitrite transporter	Chromosome1
gene4703	narK	K02575	MFS transporter, NNP family, nitrate/nitrite transporter	Chromosome2
gene5230	narK	K02575	MFS transporter, NNP family, nitrate/nitrite transporter	Chromosome2
gene4702	nirB	K00362	nitrite reductase (NADH) large subunit [EC:1.7.1.15]	Chromosome2
gene0596	gdhA	K00261	glutamate dehydrogenase (NAD(P)+) [EC:1.4.1.3]	Chromosome1
gene2682	iscU	K04488	nitrogen fixation protein NifU and related proteins	Chromosome1
gene2715	glnA	K01915	glutamine synthetase [EC:6.3.1.2]	Chromosome1
gene2729	glnA	K01915	glutamine synthetase [EC:6.3.1.2]	Chromosome1
gene3034	glnB	K04751	nitrogen regulatory protein P-II 1	Chromosome1
gene3867	cynS	K01725	cyanate lyase [EC:4.2.1.104]	Chromosome2
gene4726	cynS	K01725	cyanate lyase [EC:4.2.1.104]	Chromosome2
gene2837	cynT	K01673	carbonic anhydrase [EC:4.2.1.1]	Chromosome1
gene2838	cynT	K01673	carbonic anhydrase [EC:4.2.1.1]	Chromosome1
gene3542	cynT	K01673	carbonic anhydrase [EC:4.2.1.1]	Chromosome1
gene4725	cynT	K01673	carbonic anhydrase [EC:4.2.1.1]	Chromosome2

Table S5. Genes associated with zinc solubilization in the complete genome of *Burkholderia gladioli* YNK-FB0053

Gene ID	Gene name	KO ID	KO Description	Location
gene3220	zur	K09823	Fur family transcriptional regulator, zinc uptake regulator	Chromosome1
gene6262	zur	K09823	Fur family transcriptional regulator, zinc uptake regulator	Chromosome2
gene0106	zntA	K01534	Zn2+/Cd2+-exporting ATPase [EC:7.2.2.12 7.2.2.21]	Chromosome1
gene2062	zntA	K01534	Zn2+/Cd2+-exporting ATPase [EC:7.2.2.12 7.2.2.21]	Chromosome1
gene0419	zntB	K16074	zinc transporter	Chromosome1
gene0899	tonB	K03832	periplasmic protein TonB	Chromosome1
gene2771	tonB	K03832	periplasmic protein TonB	Chromosome1
gene3258	tonB	K03832	periplasmic protein TonB	Chromosome1
gene4967	tonB	K03832	periplasmic protein TonB	Chromosome2
gene6049	tonB	K03832	periplasmic protein TonB	Chromosome2
gene6337	tonB	K03832	periplasmic protein TonB	Chromosome2
gene0580	exbB	K03561	biopolymer transport protein ExbB	Chromosome1
gene2772	exbB	K03561	biopolymer transport protein ExbB	Chromosome1
gene6048	exbB	K03561	biopolymer transport protein ExbB	Chromosome2
gene6338	exbB	K03561	biopolymer transport protein ExbB	Chromosome2
gene0581	exbD	K03559	biopolymer transport protein ExbD	Chromosome1
gene2773	exbD	K03559	biopolymer transport protein ExbD	Chromosome1
gene6046	exbD	K03559	biopolymer transport protein ExbD	Chromosome2
gene6047	exbD	K03559	biopolymer transport protein ExbD	Chromosome2
gene6339	exbD	K03559	biopolymer transport protein ExbD	Chromosome2

Table S6. Genes associated with siderophore biosynthesis and iron transport in the complete genome of *Burkholderia gladioli* YNK-FB0053

Gene ID	Gene name	KO ID	KO Description	Location
gene5237	entS	K08225	MFS transporter, ENTS family, enterobactin (siderophore) exporter	Chromosome2
gene5284	afuA	K02012	iron(III) transport system substrate-binding protein	Chromosome2
gene5319	afuA	K02012	iron(III) transport system substrate-binding protein	Chromosome2
gene6842	afuA	K02012	iron(III) transport system substrate-binding protein	Chromosome2
gene5285	afuB	K02011	iron(III) transport system permease protein	Chromosome2
gene5320	afuB	K02011	iron(III) transport system permease protein	Chromosome2
gene6840	afuB	K02011	iron(III) transport system permease protein	Chromosome2
gene5286	afuC	K02010	iron(III) transport system ATP-binding protein [EC:7.2.2.7]	Chromosome2
gene5318	afuC	K02010	iron(III) transport system ATP-binding protein [EC:7.2.2.7]	Chromosome2
gene6841	afuC	K02010	iron(III) transport system ATP-binding protein [EC:7.2.2.7]	Chromosome2
gene0577	furB	K03711	Fur family transcriptional regulator, ferric uptake regulator	Chromosome1
gene4693	furB	K03711	Fur family transcriptional regulator, ferric uptake regulator	Chromosome2
gene5464	furB	K03711	Fur family transcriptional regulator, ferric uptake regulator	Chromosome2

gene6606	furB	K03711	Fur family transcriptional regulator, ferric uptake regulator	Chromosome2
gene2780	efeU	K07243	high-affinity iron transporter	Chromosome1
gene1353	ftrA	K13633	AraC family transcriptional regulator, transcriptional activator FtrA	Chromosome1
gene1871	ftrA	K13633	AraC family transcriptional regulator, transcriptional activator FtrA	Chromosome1
gene2429	ftrA	K13633	AraC family transcriptional regulator, transcriptional activator FtrA	Chromosome1
gene3900	ftrA	K13633	AraC family transcriptional regulator, transcriptional activator FtrA	Chromosome2
gene5299	ftrA	K13633	AraC family transcriptional regulator, transcriptional activator FtrA	Chromosome2
gene5726	ftrA	K13633	AraC family transcriptional regulator, transcriptional activator FtrA	Chromosome2
gene2782	ftrA	K07230	periplasmic iron binding protein	Chromosome1

Table S7. Genes associated with indole-3-acetic acid (IAA) biosynthesis in the complete genome of *Burkholderia gladioli* YNK-FB0053

Gene ID	Gene name	KO ID	KO Description	Location
gene0468	trpC	K01609	indole-3-glycerol phosphate synthase [EC:4.1.1.48]	Chromosome1
gene0469	trpD	K00766	anthranilate phosphoribosyltransferase [EC:2.4.2.18]	Chromosome1
gene3431	trpD	K00766	anthranilate phosphoribosyltransferase	Chromosome1

			[EC:2.4.2.18]	
gene0470	trpG	K01658	anthranilate synthase component II [EC:4.1.3.27]	Chromosome1
gene1494	trpG	K01658	anthranilate synthase component II [EC:4.1.3.27]	Chromosome1
gene0471	trpE	K01657	anthranilate synthase component I [EC:4.1.3.27]	Chromosome1
gene2654	trpS	K01867	tryptophanyl-tRNA synthetase [EC:6.1.1.2]	Chromosome1
gene6627	trpS	K01867	tryptophanyl-tRNA synthetase [EC:6.1.1.2]	Chromosome2
gene4831	trpA	K01695	tryptophan synthase alpha chain [EC:4.2.1.20]	Chromosome2
gene4833	trpB	K01696	tryptophan synthase beta chain [EC:4.2.1.20]	Chromosome2
gene4834	trpF	K01817	phosphoribosylanthranilate isomerase [EC:5.3.1.24]	Chromosome2
gene0479	ALDH	K00128	aldehyde dehydrogenase (NAD+) [EC:1.2.1.3]	Chromosome1
gene0851	ALDH	K00128	aldehyde dehydrogenase (NAD+) [EC:1.2.1.3]	Chromosome1
gene6018	ALDH	K00128	aldehyde dehydrogenase (NAD+) [EC:1.2.1.3]	Chromosome2
gene1560	ALDH	K00128	aldehyde dehydrogenase (NAD+) [EC:1.2.1.3]	Chromosome1
gene3039	ALDH	K00128	aldehyde dehydrogenase (NAD+) [EC:1.2.1.3]	Chromosome1
gene3943	ALDH	K00128	aldehyde dehydrogenase (NAD+) [EC:1.2.1.3]	Chromosome2
gene4083	ALDH	K00128	aldehyde dehydrogenase (NAD+) [EC:1.2.1.3]	Chromosome2
gene5176	ALDH	K00128	aldehyde dehydrogenase (NAD+) [EC:1.2.1.3]	Chromosome2
gene5591	ALDH	K00128	aldehyde dehydrogenase (NAD+) [EC:1.2.1.3]	Chromosome2
gene6887	ALDH	K00128	aldehyde dehydrogenase (NAD+) [EC:1.2.1.3]	Chromosome2
gene1094	amiE	K01426	amidase [EC:3.5.1.4]	Chromosome1
gene2590	amiE	K01426	amidase [EC:3.5.1.4]	Chromosome1

gene2600	amiE	K01426	amidase [EC:3.5.1.4]	Chromosome1
gene3952	amiE	K01426	amidase [EC:3.5.1.4]	Chromosome2
gene4093	amiE	K01426	amidase [EC:3.5.1.4]	Chromosome2
gene6679	amiE	K01426	amidase [EC:3.5.1.4]	Chromosome2

Table S8. Genes associated with phenolic acid degradation in the complete genome of *Burkholderia gladioli* YNK-FB0053

Gene ID	Gene name	KO ID	KO Description	Location
gene4803	benA-xylX	K05549	benzoate/toluate 1,2-dioxygenase subunit alpha [EC:1.14.12.10 1.14.12.-]	Chromosome2
gene4802	benB-xylY	K05550	benzoate/toluate 1,2-dioxygenase subunit beta [EC:1.14.12.10 1.14.12.-]	Chromosome2
gene4801	benC-xylZ	K05784	benzoate/toluate 1,2-dioxygenase reductase component [EC:1.18.1.-]	Chromosome2
gene4800	benD-xylL	K05783	dihydroxycyclohexadiene carboxylate dehydrogenase [EC:1.3.1.25 1.3.1.-]	Chromosome2
gene2923	benE	K05782	benzoate membrane transport protein	Chromosome1
gene1294	benM	K21757	LysR family transcriptional regulator, benzoate and cis,cis-muconate-responsive activator of ben and cat genes	Chromosome1
gene3080	benM	K21757	LysR family transcriptional regulator, benzoate and cis,cis-muconate-responsive activator of ben and cat genes	Chromosome1
gene3357	benM	K21757	LysR family transcriptional regulator, benzoate and cis,cis-muconate-responsive activator of ben and cat genes	Chromosome1
gene4041	benM	K21757	LysR family transcriptional regulator, benzoate and cis,cis-muconate-responsive activator of ben and cat genes	Chromosome2
gene4223	benM	K21757	LysR family transcriptional regulator, benzoate and cis,cis-muconate-responsive activator of ben and cat genes	Chromosome2

gene5870	benM	K21757	LysR family transcriptional regulator, benzoate and cis,cis-muconate-responsive activator of ben and cat genes	Chromosome2
gene6509	benM	K21757	LysR family transcriptional regulator, benzoate and cis,cis-muconate-responsive activator of ben and cat genes	Chromosome2
gene2365	benM	K21757	LysR family transcriptional regulator, benzoate and cis,cis-muconate-responsive activator of ben and cat genes	Chromosome1
gene2887	benM	K21757	LysR family transcriptional regulator, benzoate and cis,cis-muconate-responsive activator of ben and cat genes	Chromosome1
gene3302	benM	K21757	LysR family transcriptional regulator, benzoate and cis,cis-muconate-responsive activator of ben and cat genes	Chromosome1
gene4596	benM	K21757	LysR family transcriptional regulator, benzoate and cis,cis-muconate-responsive activator of ben and cat genes	Chromosome2
gene4799	benM	K21757	LysR family transcriptional regulator, benzoate and cis,cis-muconate-responsive activator of ben and cat genes	Chromosome2
gene5189	benM	K21757	LysR family transcriptional regulator, benzoate and cis,cis-muconate-responsive activator of ben and cat genes	Chromosome2
gene5634	benM	K21757	LysR family transcriptional regulator, benzoate and cis,cis-muconate-responsive activator of ben and cat genes	Chromosome2

gene6216	benM	K21757	LysR family transcriptional regulator, benzoate and cis,cis-muconate-responsive activator of ben and cat genes	Chromosome2
gene5191	catA	K03381	catechol 1,2-dioxygenase [EC:1.13.11.1]	Chromosome2
gene5190	catB	K01856	muconate cycloisomerase [EC:5.5.1.1]	Chromosome2
gene5192	catC	K03464	muconolactone D-isomerase [EC:5.3.3.4]	Chromosome2
gene6903	catE	K07104	catechol 2,3-dioxygenase [EC:1.13.11.2]	Chromosome2
gene0744	pcaK	K08195	MFS transporter, AAHS family, 4-hydroxybenzoate transporter	Chromosome1
gene3207	pcaK	K08195	MFS transporter, AAHS family, 4-hydroxybenzoate transporter	Chromosome1
gene4237	pcaK	K08195	MFS transporter, AAHS family, 4-hydroxybenzoate transporter	Chromosome2
gene4886	pcaK	K08195	MFS transporter, AAHS family, 4-hydroxybenzoate transporter	Chromosome2
gene5767	pcaK	K08195	MFS transporter, AAHS family, 4-hydroxybenzoate transporter	Chromosome2
gene2237	pcaR	K02624	IclR family transcriptional regulator, pca regulon regulatory protein	Chromosome1
gene3529	pcaR	K02624	IclR family transcriptional regulator, pca regulon regulatory protein	Chromosome1
gene4071	pcaR	K02624	IclR family transcriptional regulator, pca regulon regulatory protein	Chromosome2
gene4079	pcaR	K02624	IclR family transcriptional regulator, pca regulon regulatory protein	Chromosome2
gene4489	pcaR	K02624	IclR family transcriptional regulator, pca	Chromosome2

			regulon regulatory protein	
gene5972	pcaR	K02624	IclR family transcriptional regulator, pca regulon regulatory protein	Chromosome2
gene6291	pcaR	K02624	IclR family transcriptional regulator, pca regulon regulatory protein	Chromosome2
gene6568	pcaR	K02624	IclR family transcriptional regulator, pca regulon regulatory protein	Chromosome2
gene6909	pcaR	K02624	IclR family transcriptional regulator, pca regulon regulatory protein	Chromosome2
gene4078	pcaD	K01055	3-oxoadipate enol-lactonase [EC:3.1.1.24]	Chromosome2
gene5435	pcaD	K01055	3-oxoadipate enol-lactonase [EC:3.1.1.24]	Chromosome2
gene6234	pcaD	K01055	3-oxoadipate enol-lactonase [EC:3.1.1.24]	Chromosome2
gene6408	pcaD	K01055	3-oxoadipate enol-lactonase [EC:3.1.1.24]	Chromosome2
gene6936	pcaD	K01055	3-oxoadipate enol-lactonase [EC:3.1.1.24]	Chromosome2
gene4120	pcaQ	K02623	LysR family transcriptional regulator, pca operon transcriptional activator	Chromosome2
gene4309	pcaQ	K02623	LysR family transcriptional regulator, pca operon transcriptional activator	Chromosome2
gene4448	pcaQ	K02623	LysR family transcriptional regulator, pca operon transcriptional activator	Chromosome2
gene4446	pcaG	K00448	protocatechuate 3,4-dioxygenase, alpha subunit [EC:1.13.11.3]	Chromosome2
gene4447	pcaH	K00449	protocatechuate 3,4-dioxygenase, beta subunit [EC:1.13.11.3]	Chromosome2
gene4741	pcaC	K01607	4-carboxymuconolactone decarboxylase [EC:4.1.1.44]	Chromosome2

gene5041	pcaC	K01607	4-carboxymuconolactone decarboxylase [EC:4.1.1.44]	Chromosome2
gene5693	pcaC	K01607	4-carboxymuconolactone decarboxylase [EC:4.1.1.44]	Chromosome2
gene6067	pcaC	K01607	4-carboxymuconolactone decarboxylase [EC:4.1.1.44]	Chromosome2
gene6235	pcaC	K01607	4-carboxymuconolactone decarboxylase [EC:4.1.1.44]	Chromosome2
gene6231	pcaI	K01031	3-oxoadipate CoA-transferase, alpha subunit [EC:2.8.3.6]	Chromosome2
gene6232	pcaJ	K01032	3-oxoadipate CoA-transferase, beta subunit [EC:2.8.3.6]	Chromosome2
gene6233	pcaB	K01857	3-carboxy-cis,cis-muconate cycloisomerase [EC:5.5.1.2]	Chromosome2
gene6569	pcaF	K07823	3-oxoadipyl-CoA thiolase [EC:2.3.1.174]	Chromosome2
gene3350	fadA	K00632	acetyl-CoA acyltransferase [EC:2.3.1.16]	Chromosome1
gene0487	fadD	K01897	long-chain acyl-CoA synthetase [EC:6.2.1.3]	Chromosome1
gene1685	fadD	K01897	long-chain acyl-CoA synthetase [EC:6.2.1.3]	Chromosome1
gene3188	fadD	K01897	long-chain acyl-CoA synthetase [EC:6.2.1.3]	Chromosome1
gene5620	fadD	K01897	long-chain acyl-CoA synthetase [EC:6.2.1.3]	Chromosome2
gene1948	fadH	K00219	2,4-dienoyl-CoA reductase (NADPH2) [EC:1.3.1.34]	Chromosome1
gene1949	fadH	K00219	2,4-dienoyl-CoA reductase (NADPH2) [EC:1.3.1.34]	Chromosome1
gene5200	fadH	K00219	2,4-dienoyl-CoA reductase (NADPH2) [EC:1.3.1.34]	Chromosome2

gene2274	fadN	K07516	3-hydroxyacyl-CoA dehydrogenase [EC:1.1.1.35]	Chromosome1
gene3351	fadN	K07516	3-hydroxyacyl-CoA dehydrogenase [EC:1.1.1.35]	Chromosome1
gene3491	fadN	K07516	3-hydroxyacyl-CoA dehydrogenase [EC:1.1.1.35]	Chromosome1
gene0741	gcdH	K00252	glutaryl-CoA dehydrogenase [EC:1.3.8.6]	Chromosome1
gene0981	gcd	K00117	quinoprotein glucose dehydrogenase [EC:1.1.5.2]	Chromosome1
gene3805	gcd	K00117	quinoprotein glucose dehydrogenase [EC:1.1.5.2]	Chromosome2
gene4348	gcd	K00117	quinoprotein glucose dehydrogenase [EC:1.1.5.2]	Chromosome2
gene1555	gcdG	K01041	glutaryl-CoA transferase [EC:2.8.3.-]	Chromosome1
gene0260	paaE	K02613	ring-1,2-phenylacetyl-CoA epoxidase subunit PaaE	Chromosome1
gene0261	paaD	K02612	ring-1,2-phenylacetyl-CoA epoxidase subunit PaaD	Chromosome1
gene0262	paaC	K02611	ring-1,2-phenylacetyl-CoA epoxidase subunit PaaC [EC:1.14.13.149]	Chromosome1
gene0263	paaB	K02610	ring-1,2-phenylacetyl-CoA epoxidase subunit PaaB	Chromosome1
gene0264	paaA	K02609	ring-1,2-phenylacetyl-CoA epoxidase subunit PaaA [EC:1.14.13.149]	Chromosome1
gene0476	paaK	K01912	phenylacetate-CoA ligase [EC:6.2.1.30]	Chromosome1
gene0477	paaI	K02614	acyl-CoA thioesterase [EC:3.1.2.-]	Chromosome1

gene1773	paaI	K02614	acyl-CoA thioesterase [EC:3.1.2.-]	Chromosome1
gene2271	paaI	K02614	acyl-CoA thioesterase [EC:3.1.2.-]	Chromosome1
gene0478	paaG	K15866	2-(1,2-epoxy-1,2-dihydrophenyl)acetyl-CoA isomerase [EC:5.3.3.18]	Chromosome1
gene2804	paaG	K15866	2-(1,2-epoxy-1,2-dihydrophenyl)acetyl-CoA isomerase [EC:5.3.3.18]	Chromosome1
gene3349	paaG	K15866	2-(1,2-epoxy-1,2-dihydrophenyl)acetyl-CoA isomerase [EC:5.3.3.18]	Chromosome1
gene5061	paaG	K15866	2-(1,2-epoxy-1,2-dihydrophenyl)acetyl-CoA isomerase [EC:5.3.3.18]	Chromosome2
gene0480	paaF	K01692	enoyl-CoA hydratase [EC:4.2.1.17]	Chromosome1
gene5062	paaF	K01692	enoyl-CoA hydratase [EC:4.2.1.17]	Chromosome2
gene1097	atoB	K00626	acetyl-CoA C-acetyltransferase [EC:2.3.1.9]	Chromosome1
gene1912	atoB	K00626	acetyl-CoA C-acetyltransferase [EC:2.3.1.9]	Chromosome1
gene2160	atoB	K00626	acetyl-CoA C-acetyltransferase [EC:2.3.1.9]	Chromosome1
gene2167	atoB	K00626	acetyl-CoA C-acetyltransferase [EC:2.3.1.9]	Chromosome1
gene2272	atoB	K00626	acetyl-CoA C-acetyltransferase [EC:2.3.1.9]	Chromosome1
gene3543	atoB	K00626	acetyl-CoA C-acetyltransferase [EC:2.3.1.9]	Chromosome1
gene3614	atoB	K00626	acetyl-CoA C-acetyltransferase [EC:2.3.1.9]	Chromosome1
gene5773	atoB	K00626	acetyl-CoA C-acetyltransferase [EC:2.3.1.9]	Chromosome2
gene1359	atoC	K07714	two-component system, NtrC family, response regulator AtoC	Chromosome1
gene5009	atoC	K07714	two-component system, NtrC family, response regulator AtoC	Chromosome2
gene5712	atoC	K07714	two-component system, NtrC family,	Chromosome2

			response regulator AtoC	
gene6179	atoC	K07714	two-component system, NtrC family, response regulator AtoC	Chromosome2
gene3864	atoE	K02106	short-chain fatty acids transporter	Chromosome2
gene4766	atoE	K02106	short-chain fatty acids transporter	Chromosome2
gene1055	nagZ	K01207	beta-N-acetylhexosaminidase [EC:3.2.1.52]	Chromosome1
gene2366	nagA	K01443	N-acetylglucosamine-6-phosphate deacetylase [EC:3.5.1.25]	Chromosome1
gene3443	nagA	K01443	N-acetylglucosamine-6-phosphate deacetylase [EC:3.5.1.25]	Chromosome1
gene3160	nagR	K24967	GntR family transcriptional regulator, N-acetylglucosamine utilization regulator	Chromosome1
gene3430	nagL	K01801	maleylpyruvate isomerase [EC:5.2.1.4]	Chromosome1
gene3440	nagE	K02804	N-acetylglucosamine PTS system EIICBA or EIICB component [EC:2.7.1.193]	Chromosome1
gene4431	nagK	K16165	fumarylpyruvate hydrolase [EC:3.7.1.20]	Chromosome2

Table S9. Genes associated with bacterial chemotaxis in the complete genome of *Burkholderia gladioli* YNK-FB0053

Gene ID	Gene name	KO ID	KO Description	Location
gene0199	tsr	K05874	methyl-accepting chemotaxis protein I, serine sensor receptor	Chromosome1
gene3856	tsr	K05874	methyl-accepting chemotaxis protein I, serine sensor receptor	Chromosome2

gene3902	tsr	K05874	methyl-accepting chemotaxis protein I, serine sensor receptor	Chromosome2
gene4131	tsr	K05874	methyl-accepting chemotaxis protein I, serine sensor receptor	Chromosome2
gene4723	tsr	K05874	methyl-accepting chemotaxis protein I, serine sensor receptor	Chromosome2
gene4936	tsr	K05874	methyl-accepting chemotaxis protein I, serine sensor receptor	Chromosome2
gene5474	tsr	K05874	methyl-accepting chemotaxis protein I, serine sensor receptor	Chromosome2
gene6449	tsr	K05874	methyl-accepting chemotaxis protein I, serine sensor receptor	Chromosome2
gene6931	tsr	K05874	methyl-accepting chemotaxis protein I, serine sensor receptor	Chromosome2
gene0168	mcp	K03406	methyl-accepting chemotaxis protein	Chromosome1
gene1098	mcp	K03406	methyl-accepting chemotaxis protein	Chromosome1
gene1452	mcp	K03406	methyl-accepting chemotaxis protein	Chromosome1
gene1599	mcp	K03406	methyl-accepting chemotaxis protein	Chromosome1
gene1849	mcp	K03406	methyl-accepting chemotaxis protein	Chromosome1
gene2550	mcp	K03406	methyl-accepting chemotaxis protein	Chromosome1
gene3147	mcp	K03406	methyl-accepting chemotaxis protein	Chromosome1
gene3280	mcp	K03406	methyl-accepting chemotaxis protein	Chromosome1
gene4113	mcp	K03406	methyl-accepting chemotaxis protein	Chromosome2

gene4408	mcp	K03406	methyl-accepting chemotaxis protein	Chromosome2
gene4510	mcp	K03406	methyl-accepting chemotaxis protein	Chromosome2
gene5361	mcp	K03406	methyl-accepting chemotaxis protein	Chromosome2
gene5783	mcp	K03406	methyl-accepting chemotaxis protein	Chromosome2
gene6346	mcp	K03406	methyl-accepting chemotaxis protein	Chromosome2
gene6845	mcp	K03406	methyl-accepting chemotaxis protein	Chromosome2
gene6867	mcp	K03406	methyl-accepting chemotaxis protein	Chromosome2
gene6877	mcp	K03406	methyl-accepting chemotaxis protein	Chromosome2
gene4975	aer	K03776	aerotaxis receptor	Chromosome2
gene6572	aer	K03776	aerotaxis receptor	Chromosome2
gene0198	cheW	K03408	purine-binding chemotaxis protein CheW	Chromosome1
gene0197	cheA	K03407	two-component system, chemotaxis family, sensor kinase CheA [EC:2.7.13.3]	Chromosome1
gene3148	cheA	K03407	two-component system, chemotaxis family, sensor kinase CheA [EC:2.7.13.3]	Chromosome1
gene5717	cheA	K03407	two-component system, chemotaxis family, sensor kinase CheA [EC:2.7.13.3]	Chromosome2
gene0196	cheY	K03413	two-component system, chemotaxis family, chemotaxis protein CheY	Chromosome1
gene0203	cheY	K03413	two-component system, chemotaxis family, chemotaxis protein CheY	Chromosome1
gene3144	cheY	K03413	two-component system, chemotaxis family, chemotaxis protein CheY	Chromosome1

gene0198	cheW	K03408	purine-binding chemotaxis protein CheW	Chromosome1
gene0200	cheR	K00575	chemotaxis protein methyltransferase CheR [EC:2.1.1.80]	Chromosome1
gene5716	cheR	K00575	chemotaxis protein methyltransferase CheR [EC:2.1.1.80]	Chromosome2
gene0201	cheD	K03411	chemotaxis protein CheD [EC:3.5.1.44]	Chromosome1
gene0202	cheB	K03412	two-component system, chemotaxis family, protein-glutamate methylesterase/glutaminase [EC:3.1.1.61 3.5.1.44]	Chromosome1
gene5715	cheB	K03412	two-component system, chemotaxis family, protein-glutamate methylesterase/glutaminase [EC:3.1.1.61 3.5.1.44]	Chromosome2
gene0204	cheZ	K03414	chemotaxis protein CheZ	Chromosome1
gene1360	cheBR	K13924	two-component system, chemotaxis family, CheB/CheR fusion protein [EC:2.1.1.80 3.1.1.61]	Chromosome1
gene5107	cheBR	K13924	two-component system, chemotaxis family, CheB/CheR fusion protein [EC:2.1.1.80 3.1.1.61]	Chromosome2
gene5781	cheBR	K13924	two-component system, chemotaxis family, CheB/CheR fusion protein [EC:2.1.1.80 3.1.1.61]	Chromosome2
gene6445	cheV	K03415	two-component system, chemotaxis family, chemotaxis protein CheV	Chromosome2
gene0026	fliR	K02421	flagellar biosynthesis protein FliR	Chromosome1
gene0027	fliQ	K02420	flagellar biosynthesis protein FliQ	Chromosome1
gene0028	fliP	K02419	flagellar biosynthesis protein FliP	Chromosome1

gene0029	fliO	K02418	flagellar protein FliO/FliZ	Chromosome1
gene0030	fliN	K02417	flagellar motor switch protein FliN	Chromosome1
gene0031	fliM	K02416	flagellar motor switch protein FliM	Chromosome1
gene0032	fliL	K02415	flagellar protein FliL	Chromosome1
gene6926	fliL	K02415	flagellar protein FliL	Chromosome2
gene0183	fliT	K02423	flagellar protein FliT	Chromosome1
gene3688	fliT	K02423	flagellar protein FliT	Chromosome1
gene0184	fliD	K02407	flagellar hook-associated protein 2	Chromosome1
gene0185	fliC	K02406	flagellin	Chromosome1
gene0212	fliA	K02405	RNA polymerase sigma factor FliA	Chromosome1
gene3689	fliS	K02422	flagellar secretion chaperone FliS	Chromosome1
gene3690	fliE	K02408	flagellar hook-basal body complex protein FliE	Chromosome1
gene3691	fliF	K02409	flagellar M-ring protein FliF	Chromosome1
gene3692	fliG	K02410	flagellar motor switch protein FliG	Chromosome1
gene3693	fliH	K02411	flagellar assembly protein FliH	Chromosome1
gene3694	fliI	K02412	flagellum-specific ATP synthase [EC:7.4.2.8]	Chromosome1
gene3695	fliJ	K02413	flagellar protein FliJ	Chromosome1
gene3696	fliK	K02414	flagellar hook-length control protein FliK	Chromosome1

Table S10. Genes associated with flagellar biosynthesis and assembly in the complete genome of *Burkholderia gladioli* YNK-FB0053

Gene ID	Gene name	KO ID	KO Description	Location

gene0194	motA	K02556	chemotaxis protein MotA	Chromosome1
gene4979	motA	K02556	chemotaxis protein MotA	Chromosome2
gene0195	motB	K02557	chemotaxis protein MotB	Chromosome1
gene4760	motB	K02557	chemotaxis protein MotB	Chromosome2
gene4980	motB	K02557	chemotaxis protein MotB	Chromosome2
gene0026	fliR	K02421	flagellar biosynthesis protein FliR	Chromosome1
gene0027	fliQ	K02420	flagellar biosynthesis protein FliQ	Chromosome1
gene0028	fliP	K02419	flagellar biosynthesis protein FliP	Chromosome1
gene0029	fliO	K02418	flagellar protein FliO/FliZ	Chromosome1
gene0030	fliN	K02417	flagellar motor switch protein FliN	Chromosome1
gene0031	fliM	K02416	flagellar motor switch protein FliM	Chromosome1
gene0032	fliL	K02415	flagellar protein FliL	Chromosome1
gene6926	fliL	K02415	flagellar protein FliL	Chromosome2
gene0183	fliT	K02423	flagellar protein FliT	Chromosome1
gene3688	fliT	K02423	flagellar protein FliT	Chromosome1
gene0184	fliD	K02407	flagellar hook-associated protein 2	Chromosome1
gene0185	fliC	K02406	flagellin	Chromosome1
gene0212	fliA	K02405	RNA polymerase sigma factor FliA	Chromosome1
gene3689	fliS	K02422	flagellar secretion chaperone FliS	Chromosome1
gene3690	fliE	K02408	flagellar hook-basal body complex protein FliE	Chromosome1
gene3691	fliF	K02409	flagellar M-ring protein FliF	Chromosome1
gene3692	fliG	K02410	flagellar motor switch protein FliG	Chromosome1
gene3693	fliH	K02411	flagellar assembly protein FliH	Chromosome1
gene3694	fliI	K02412	flagellum-specific ATP synthase [EC:7.4.2.8]	Chromosome1

gene3695	fliJ	K02413	flagellar protein FliJ	Chromosome1
gene3696	fliK	K02414	flagellar hook-length control protein FliK	Chromosome1
gene0999	flgR	K02481	two-component system, NtrC family, response regulator	Chromosome1
gene2044	flgR	K02481	two-component system, NtrC family, response regulator	Chromosome1
gene4450	flgR	K02481	two-component system, NtrC family, response regulator	Chromosome2
gene6922	flgR	K02481	two-component system, NtrC family, response regulator	Chromosome2
gene2017	flgS	K02482	two-component system, NtrC family, sensor kinase [EC:2.7.13.3]	Chromosome1
gene6075	flgS	K02482	two-component system, NtrC family, sensor kinase [EC:2.7.13.3]	Chromosome2
gene3645	flgL	K02397	flagellar hook-associated protein 3 FlgL	Chromosome1
gene3646	flgK	K02396	flagellar hook-associated protein 1	Chromosome1
gene3648	flgJ	K02395	peptidoglycan hydrolase FlgJ	Chromosome1
gene3925	flgJ	K02395	peptidoglycan hydrolase FlgJ	Chromosome2
gene3649	flgI	K02394	flagellar P-ring protein FlgI	Chromosome1
gene3650	flgH	K02393	flagellar L-ring protein FlgH	Chromosome1
gene3651	flgG	K02392	flagellar basal-body rod protein FlgG	Chromosome1
gene3652	flgF	K02391	flagellar basal-body rod protein FlgF	Chromosome1
gene3653	flgE	K02390	flagellar hook protein FlgE	Chromosome1
gene3654	flgD	K02389	flagellar basal-body rod modification protein FlgD	Chromosome1

gene3655	flgC	K02388	flagellar basal-body rod protein FlgC	Chromosome1
gene3656	flgB	K02387	flagellar basal-body rod protein FlgB	Chromosome1
gene3657	flgA	K02386	flagellar basal body P-ring formation protein FlgA	Chromosome1
gene3658	flgM	K02398	negative regulator of flagellin synthesis FlgM	Chromosome1
gene3659	flgN	K02399	flagellar biosynthesis protein FlgN	Chromosome1
gene0192	flhD	K02403	flagellar transcriptional activator FlhD	Chromosome1
gene6865	flhD	K02403	flagellar transcriptional activator FlhD	Chromosome2
gene0193	flhC	K02402	flagellar transcriptional activator FlhC	Chromosome1
gene0208	flhB	K02401	flagellar biosynthesis protein FlhB	Chromosome1
gene0209	flhA	K02400	flagellar biosynthesis protein FlhA	Chromosome1
gene0210	flhF	K02404	flagellar biosynthesis protein FlhF	Chromosome1
gene0211	flhG	K04562	flagellar biosynthesis protein FlhG	Chromosome1
gene3686	flhB2	K04061	flagellar biosynthesis protein	Chromosome1

Table S11. Genes associated with exopolysaccharide biosynthesis in the complete genome of *Burkholderia gladioli* YNK-FB0053

Gene ID	Gene name	KO ID	KO Description	Location
gene2580	cysE	K00640	serine O-acetyltransferase [EC:2.3.1.30]	Chromosome1
gene6226	cysE	K00640	serine O-acetyltransferase [EC:2.3.1.30]	Chromosome2
gene1226	pgaC	K11936	poly-beta-1,6-N-acetyl-D-glucosamine synthase [EC:2.4.1.-]	Chromosome1
gene2005	pgaB	K11931	poly-beta-1,6-N-acetyl-D-glucosamine N-deacetylase [EC:3.5.1.-]	Chromosome1

gene5140	gumD	K13656	undecaprenyl-phosphate glucose phosphotransferase [EC:2.7.8.31]	Chromosome2

Table S12. Genes associated with copper stress in the complete genome of *Burkholderia gladioli* YNK-FB0053

Gene ID	Gene name	KO ID	KO Description	Location
gene4012	cusR	K07665	two-component system, OmpR family, copper resistance phosphate regulon response regulator CusR	Chromosome2
gene4459	cusR	K07665	two-component system, OmpR family, copper resistance phosphate regulon response regulator CusR	Chromosome2
gene5417	cusR	K07665	two-component system, OmpR family, copper resistance phosphate regulon response regulator CusR	Chromosome2
gene5877	cusR	K07665	two-component system, OmpR family, copper resistance phosphate regulon response regulator CusR	Chromosome2
gene6369	cusR	K07665	two-component system, OmpR family, copper resistance phosphate regulon response regulator CusR	Chromosome2
gene6618	cusR	K07665	two-component system, OmpR family, copper resistance phosphate regulon response regulator CusR	Chromosome2
gene4013	cusS	K07644	two-component system, OmpR family, heavy metal sensor histidine kinase CusS [EC:2.7.13.3]	Chromosome2
gene4458	cusS	K07644	two-component system, OmpR family, heavy metal sensor histidine kinase CusS [EC:2.7.13.3]	Chromosome2
gene5876	cusS	K07644	two-component system, OmpR family, heavy metal sensor histidine kinase CusS [EC:2.7.13.3]	Chromosome2

gene6617	cusS	K07644	two-component system, OmpR family, heavy metal sensor histidine kinase CusS [EC:2.7.13.3]	Chromosome2
gene4895	cusB	K07798	membrane fusion protein, copper/silver efflux system	Chromosome2
gene4896	cusA	K07787	copper/silver efflux system protein	Chromosome2
gene4897	cusF	K07810	Cu(I)/Ag(I) efflux system periplasmic protein CusF	Chromosome2
gene5948	copA	K17686	P-type Cu+ transporter [EC:7.2.2.8]	Chromosome2
gene3178	copB	K01533	P-type Cu2+ transporter [EC:7.2.2.9]	Chromosome1
gene3963	copC	K07156	copper resistance protein C	Chromosome2
gene6621	copC	K07156	copper resistance protein C	Chromosome2

Table S13. Genes associated with cobalt/zinc/cadmium heavy metal stress in the complete genome of *Burkholderia gladioli* YNK-FB0053

Gene ID	Gene name	KO ID	KO Description	Location
gene2038	czcA	K15726	heavy metal efflux system protein	Chromosome1
gene2135	czcA	K15726	heavy metal efflux system protein	Chromosome1
gene4009	czcA	K15726	heavy metal efflux system protein	Chromosome2
gene4010	czcB	K15727	membrane fusion protein, heavy metal efflux system	Chromosome2
gene4011	czcC	K15725	outer membrane protein, heavy metal efflux system	Chromosome2
gene4894	czcC	K15725	outer membrane protein, heavy metal efflux system	Chromosome2
gene1621	czcD	K16264	cobalt-zinc-cadmium efflux system protein	Chromosome1
gene4233	czcD	K16264	cobalt-zinc-cadmium efflux system protein	Chromosome2
gene6689	czcD	K16264	cobalt-zinc-cadmium efflux system protein	Chromosome2

Table S14. Genes associated with arsenic stress in the complete genome of *Burkholderia gladioli* YNK-FB0053

Gene ID	Gene name	KO ID	KO Description	Location
gene0085	arsB	K03893	arsenical pump membrane protein	Chromosome1
gene6930	arsB	K03893	arsenical pump membrane protein	Chromosome2
gene2482	arsC	K00537	arsenate reductase (glutaredoxin) [EC:1.20.4.1]	Chromosome1
gene3559	arsC	K00537	arsenate reductase (glutaredoxin) [EC:1.20.4.1]	Chromosome1
gene6077	arsC	K00537	arsenate reductase (glutaredoxin) [EC:1.20.4.1]	Chromosome2
gene3557	arsR	K03892	ArsR family transcriptional regulator,	Chromosome1

			arsenate/arsenite/antimonite-responsive transcriptional repressor	
gene4008	arsR	K03892	ArsR family transcriptional regulator, arsenate/arsenite/antimonite-responsive transcriptional repressor	Chromosome2
gene5328	arsR	K03892	ArsR family transcriptional regulator, arsenate/arsenite/antimonite-responsive transcriptional repressor	Chromosome2
gene3558	arsB	K03325	arsenite transporter	Chromosome1
gene3560	arsH	K11811	arsenical resistance protein ArsH	Chromosome1

Table S15. Genes associated with chromium stress in the complete genome of *Burkholderia gladioli* YNK-FB0053

Gene ID	Gene name	KO ID	KO Description	Location
gene2939	chrA	K07240	chromate transporter	Chromosome1
gene3641	chrA	K07240	chromate transporter	Chromosome1
gene3642	chrA	K07240	chromate transporter	Chromosome1
gene5999	chrA	K07240	chromate transporter	Chromosome2
gene6000	chrA	K07240	chromate transporter	Chromosome2
gene6891	chrA	K07240	chromate transporter	Chromosome2
gene6076	chrR	K19784	chromate reductase, NAD(P)H dehydrogenase (quinone)	Chromosome2
gene2939	chrA	K07240	chromate transporter	Chromosome1

Table S16. Antibiotic resistance-related genes in the complete genome of *Burkholderia gladioli* YNK-FB0053

Gene ID	Gene name	KO ID	KO Description	Location
gene1094	amiE	K01426	amidase [EC:3.5.1.4]	Chromosome1
gene2590	amiE	K01426	amidase [EC:3.5.1.4]	Chromosome1
gene2600	amiE	K01426	amidase [EC:3.5.1.4]	Chromosome1
gene3952	amiE	K01426	amidase [EC:3.5.1.4]	Chromosome2
gene4093	amiE	K01426	amidase [EC:3.5.1.4]	Chromosome2
gene6679	amiE	K01426	amidase [EC:3.5.1.4]	Chromosome2
gene1703	amiD	K11066	N-acetylmuramoyl-L-alanine amidase [EC:3.5.1.28]	Chromosome1
gene2092	amiABC	K01448	N-acetylmuramoyl-L-alanine amidase [EC:3.5.1.28]	Chromosome1
gene3193	amiABC	K01448	N-acetylmuramoyl-L-alanine amidase [EC:3.5.1.28]	Chromosome1
gene1055	nagZ	K01207	beta-N-acetylhexosaminidase [EC:3.2.1.52]	Chromosome1
gene2366	nagA	K01443	N-acetylglucosamine-6-phosphate deacetylase [EC:3.5.1.25]	Chromosome1
gene3443	nagA	K01443	N-acetylglucosamine-6-phosphate deacetylase [EC:3.5.1.25]	Chromosome1
gene3160	nagR	K24967	GntR family transcriptional regulator, N-acetylglucosamine utilization regulator	Chromosome1
gene3430	nagL	K01801	maleylpyruvate isomerase [EC:5.2.1.4]	Chromosome1
gene3440	nagE	K02804	N-acetylglucosamine PTS system EIICBA or EIICB component [EC:2.7.1.193]	Chromosome1
gene4431	nagK	K16165	fumarylpyruvate hydrolase [EC:3.7.1.20]	Chromosome2
gene5075	dnaG	K02316	DNA primase [EC:2.7.7.101]	Chromosome2
gene0501	ddl	K01921	D-alanine-D-alanine ligase [EC:6.3.2.4]	Chromosome1
gene0370	murA	K00790	UDP-N-acetylglucosamine 1-carboxyvinyltransferase [EC:2.5.1.7]	Chromosome1

gene0371	murA	K00790	UDP-N-acetylglucosamine 1-carboxyvinyltransferase [EC:2.5.1.7]	Chromosome1
gene4417	murA	K00790	UDP-N-acetylglucosamine 1-carboxyvinyltransferase [EC:2.5.1.7]	Chromosome2
gene0494	murE	K01928	UDP-N-acetylmuramoyl-L-alanyl-D-glutamate--2,6-diaminopimelate ligase [EC:6.3.2.13]	Chromosome1
gene0495	murF	K01929	UDP-N-acetylmuramoyl-tripeptide--D-alanyl-D-alanine ligase [EC:6.3.2.10]	Chromosome1
gene0497	murD	K01925	UDP-N-acetylmuramoylalanine--D-glutamate ligase [EC:6.3.2.9]	Chromosome1
gene0499	murG	K02563	UDP-N-acetylglucosamine--N-acetylmuramyl-(pentapeptide) pyrophosphoryl-undecaprenol N-acetylglucosamine transferase [EC:2.4.1.227]	Chromosome1
gene0500	murC	K01924	UDP-N-acetylmuramate--alanine ligase [EC:6.3.2.8]	Chromosome1
gene1583	murR	K15835	RpiR family transcriptional regulator, murPQ operon repressor	Chromosome1
gene6751	murR	K15835	RpiR family transcriptional regulator, murPQ operon repressor	Chromosome2
gene2769	murI	K01776	glutamate racemase [EC:5.1.1.3]	Chromosome1
gene3180	murJ	K03980	putative peptidoglycan lipid II flippase	Chromosome1
gene3184	murB	K00075	UDP-N-acetylmuramate dehydrogenase [EC:1.3.1.98]	Chromosome1
gene3344	murU	K00992	N-acetyl-alpha-D-muramate 1-phosphate uridylyltransferase [EC:2.7.7.99]	Chromosome1
gene2490	alr	K01775	alanine racemase [EC:5.1.1.1]	Chromosome1
gene5265	alr	K01775	alanine racemase [EC:5.1.1.1]	Chromosome2
gene2102	mprF	K14205	phosphatidylglycerol lysyltransferase [EC:2.3.2.3]	Chromosome1
gene3633	mprB	K07653	two-component system, OmpR family, sensor histidine kinase MprB [EC:2.7.13.3]	Chromosome1
gene2690	pbpG	K07262	serine-type D-Ala-D-Ala endopeptidase (penicillin-binding protein 7) [EC:3.4.21.-]	Chromosome1

gene3002	pbpG	K07262	serine-type D-Ala-D-Ala endopeptidase (penicillin-binding protein 7) [EC:3.4.21.-]	Chromosome1
gene4414	pbpC	K05367	penicillin-binding protein 1C [EC:2.4.1.129]	Chromosome2
gene5667	oppA	K15580	oligopeptide transport system substrate-binding protein	Chromosome2
gene5668	oppB	K15581	oligopeptide transport system permease protein	Chromosome2
gene5669	oppC	K15582	oligopeptide transport system permease protein	Chromosome2
gene5670	oppD	K15583	oligopeptide transport system ATP-binding protein	Chromosome2
gene5671	oppF	K10823	oligopeptide transport system ATP-binding protein	Chromosome2
gene0387	degQ	K04772	serine protease DegQ [EC:3.4.21.-]	Chromosome1
gene1046	degP	K04771	serine protease Do [EC:3.4.21.107]	Chromosome1
gene3179	degP	K04771	serine protease Do [EC:3.4.21.107]	Chromosome1
gene3233	degP	K04771	serine protease Do [EC:3.4.21.107]	Chromosome1
gene5380	degP	K04771	serine protease Do [EC:3.4.21.107]	Chromosome2
gene0490	mraZ	K03925	transcriptional regulator MraZ	Chromosome1
gene0491	mraW	K03438	16S rRNA (cytosine1402-N4)-methyltransferase [EC:2.1.1.199]	Chromosome1
gene0496	mraY	K01000	phospho-N-acetylmuramoyl-pentapeptide-transferase [EC:2.7.8.13]	Chromosome1
gene0370	murA	K00790	UDP-N-acetylglucosamine 1-carboxyvinyltransferase [EC:2.5.1.7]	Chromosome1
gene0371	murA	K00790	UDP-N-acetylglucosamine 1-carboxyvinyltransferase [EC:2.5.1.7]	Chromosome1
gene4417	murA	K00790	UDP-N-acetylglucosamine 1-carboxyvinyltransferase [EC:2.5.1.7]	Chromosome2
gene0494	murE	K01928	UDP-N-acetylmuramoyl-L-alanyl-D-glutamate--2,6-diaminopimelate ligase [EC:6.3.2.13]	Chromosome1
gene0495	murF	K01929	UDP-N-acetylmuramoyl-tripeptide--D-alanyl-D-alanine ligase [EC:6.3.2.10]	Chromosome1
gene0497	murD	K01925	UDP-N-acetylmuramoylalanine--D-glutamate ligase [EC:6.3.2.9]	Chromosome1
gene0499	murG	K02563	UDP-N-acetylglucosamine--N-acetylmuramyl-(pentapeptide)	Chromosome1

			pyrophosphoryl-undecaprenol N-acetylglucosamine transferase [EC:2.4.1.227]	
gene0500	murC	K01924	UDP-N-acetylmuramate--alanine ligase [EC:6.3.2.8]	Chromosome1
gene1583	murR	K15835	RpiR family transcriptional regulator, murPQ operon repressor	Chromosome1
gene6751	murR	K15835	RpiR family transcriptional regulator, murPQ operon repressor	Chromosome2
gene2769	murI	K01776	glutamate racemase [EC:5.1.1.3]	Chromosome1
gene3180	murJ	K03980	putative peptidoglycan lipid II flippase	Chromosome1
gene3184	murB	K00075	UDP-N-acetylmuramate dehydrogenase [EC:1.3.1.98]	Chromosome1
gene3344	murU	K00992	N-acetyl-alpha-D-muramate 1-phosphate uridylyltransferase [EC:2.7.7.99]	Chromosome1
gene4075	vanA	K03862	vanillate monooxygenase [EC:1.14.13.82]	Chromosome2
gene4503	vanR	K11475	GntR family transcriptional regulator, vanillate catabolism transcriptional regulator	Chromosome2
gene5032	vanR	K11475	GntR family transcriptional regulator, vanillate catabolism transcriptional regulator	Chromosome2
gene6757	vanR	K11475	GntR family transcriptional regulator, vanillate catabolism transcriptional regulator	Chromosome2
gene6123	vanB	K03863	vanillate monooxygenase ferredoxin subunit	Chromosome2
